# Proteomic analysis identifies dysregulated proteins and associated molecular pathways in a cohort of gallbladder cancer patients of African ancestry

**DOI:** 10.1186/s12014-023-09399-9

**Published:** 2023-03-01

**Authors:** Pavan Baichan, Previn Naicker, Tanya Nadine Augustine, Martin Smith, Geoffrey Candy, John Devar, Ekene Emmanuel Nweke

**Affiliations:** 1 Department of Surgery, School of Clinical Medicine, Faculty of Health Sciences, University of the Witwatersrand, 7 York Road Parktown, Johannesburg, 2193 South Africa; 2 Council for Scientific and Industrial Research, Pretoria, 0001 South Africa; 3 School of Anatomical Sciences, Faculty of Health Sciences, University of the Witwatersrand, Johannesburg, 2193 South Africa; 4 Hepatopancreatobiliary Unit, Department of Surgery, Chris Hani-Baragwanath Academic Hospital, Soweto, Johannesburg, South Africa

**Keywords:** Gallbladder cancer, Gallstone disease, Molecular pathways, Proteins, SWATH-MS

## Abstract

**Background:**

Gallbladder cancer (GBC) is a lethal cancer with a poor prognosis. The lack of specific and sensitive biomarkers results in delayed diagnosis with most patients presenting at late stages of the disease. Furthermore, there is little known about the molecular mechanisms associated with GBC, especially in patients of African ancestry. This study aimed to determine dysregulated proteins in South African GBC patients to identify potential mechanisms of the disease progression and plausible biomarkers.

**Methods:**

Tissues (27 GBC, 13 Gallstone disease, and 5 normal tissues) and blood plasma (54 GBC and 73 Benign biliary pathology) were obtained from consenting patients. Protein extraction was performed on all tissues and liquid chromatography-mass spectrometry was used for proteomic profiling. A project-specific spectral library was built using the Pulsar search algorithm. Principal component and Spearman’s rank correlation analyses were performed using PAST (V4.07b). Pathway and Network analyses were conducted using REACTOME (v3.7) and stringAPP (v1.7.0), respectively.

**Results:**

In the tissue sample group, there were 62 and 194 dysregulated proteins in GBC compared to normal and gallstone groups, respectively. In the plasma group, there were 33 altered proteins in GBC compared to the benign biliary pathology group. We found 9 proteins (APOA1, APOA2, RET4, TTR, HEMO, HBB, HBA, PIGR, and APOE) to be commonly dysregulated in both tissue and plasma. Furthermore, a subset analysis demonstrated that 2 proteins, S100A8 and S100A9, were downregulated in GBC patients with GD history compared to those without. Pathway analysis showed that the dysregulated proteins in GBC patients were enriched in pathways involved in smooth muscle contraction, metabolism, ECM organization, and integrin cell surface interactions.

**Conclusion:**

The identified dysregulated proteins help in understanding GBC molecular mechanisms in our patient group. Furthermore, the alteration of specific proteins in both tissue and plasma samples suggests their potential utility as biomarkers of GBC in this sample cohort.

**Supplementary Information:**

The online version contains supplementary material available at 10.1186/s12014-023-09399-9.

## Introduction

Gallbladder cancer (GBC) is the most prevalent cancer of the biliary tract, accounting for 80–95% of cases [1, 2]. About 80% of patients are diagnosed at an advanced or metastasised stage, hence GBC has a 5-year survival rate of ~ 19% [3]. The incidence of GBC varies with geographical location and ethnicity; with Hispanics, Bolivians, Chilean Mapuche Indians, North American Indians, and Mexican Americans appearing to have the most increased risk [4]. In 2017, there were 210,878 new cases and 173,974 deaths worldwide; consequently, the incidence and mortality of GBC increased by 76% and 65%, respectively [5]. In the United States, it is estimated that there will be 12,130 new cases and 4400 deaths in 2022 [6]. In South Africa, there were 574 histologically confirmed new cases and 287 deaths in 2018 [7]. However, a recent study evaluating records from 2003 to 2015 has suggested that there is a higher incidence of GBC in South Africa [8].

Gallbladder cancer risk factors include advanced age, female sex, gallstones, and cholecystitis [9, 10]. Clinical presentations of GBC include pain, nausea, upper right quadrant abdominal pain, jaundice, and weight loss; however, these are non-specific [11]. Due to the non-specificity of clinical presentations, GBC is characteristically diagnosed at advanced stages. This suggests a crucial need for the identification of potential biomarkers for GBC [12]. Some studies from different population groups have indicated that approximately 70–80% of GBC cases have progressed from gallstone disease (GD) history making gallstone disease a significant risk factor for GBC onset [13, 14]. However, only a small number of GD patients develop GBC, at a rate of 0.5–3%. Therefore, the molecular mechanisms linking gallstone disease to gallbladder cancer are poorly understood and hint at further scrutiny and investigation [13, 14].

Molecular changes such as protein dysregulation play a major role in GBC onset and progression [15]. Current data suggest that molecular changes associated with GBC may vary across different geographical and ethnic groups, highlighting the need for investigating these changes across the different groups [14]. Proteins are relatively stable markers; therefore, their quantification can be valuable in assessing these molecular changes in a diseased state and help identify plausible biomarkers. Ideal biomarkers are those that can circulate in the bloodstream providing a less invasive source for biomarkers of GBC [16–18]. Importantly, proteins found in both plasma and tumours may provide a convincing link that the markers are involved in tumour progression [19, 20]. Furthermore, to better understand the mechanism of onset and progression of GBC, quantified protein perturbations can be interrogated in the context of enriched biological pathways [21–24].

Liquid chromatography-mass spectrometry (LC–MS) based proteomics is a robust technique utilised for protein profiling. Sequential window of all theoretical mass spectra (SWATH-MS), a type of data-independent acquisition (DIA) LC–MS technique, also allows for reproducible analysis of prepared peptides in a systematic and unbiased manner [25, 26]. Several studies, such as the one recently conducted by our group, have demonstrated the utility of SWATH-MS in proteomic profiling in a solid tumour for the identification of potential biomarkers [27].

In this study, high-throughput SWATH-MS proteomics analysis was performed on tissue and plasma samples to identify proteomic signatures in gallbladder cancer (GBC) patients. Furthermore, by comparing the signatures in independent cohorts of tumours and plasma, we identified proteins with similar expression patterns in both sample types hinting at their biological relevance and potential utility as biomarkers. Additionally, bioinformatics analyses were used to determine the biological pathways and molecular functions of the target proteins.

## Materials and methods

### Ethics statement

Ethical approval (M190555, M160640) was obtained from the Human Research Ethics Committee of the University of the Witwatersrand, Johannesburg, South Africa. Patients provided written informed consent to be enrolled in the study.

### Sample and data collection

#### Tissues

Patients were recruited at Chris Hani Baragwanath Academic Hospital (CHBAH), Johannesburg, South Africa between April 2019 and December 2020. A total of 27 GBC tumours, 13 GD tissues, and 5 normal gallbladder tissues were used in the study. The inclusion criteria for the study were patients over 18 years of age, of African ancestry, with a clinical and histologically confirmed primary diagnosis of GBC or GD. All GBC tissues collected were identified to be advanced-stage (Stage IV) tumours according to the American Joint Committee on Cancer Staging Manual 8th Edition [28]. The exclusion criteria were patients who had an additional primary hepatopancreatobiliary disease diagnosis. A core sample of the tumour was obtained by Tru-cut ultrasound biopsy at the liver metastasis site, while the gallstone tissue was obtained via laparoscopic cholecystectomy. Non-diseased gallbladder tissues were used as normal samples and obtained from liver transplant donors. All tissues obtained were stored in approximately 700 µl of RNA*later*™ (Sigma-Aldrich, Germany) and placed in a − 80 °C freezer until further processing was required.

#### Blood

A separate cohort of patients presenting at CHBAH, Johannesburg, South Africa, were further recruited between February 2021 and October 2021. A total of 54 GBC and 73 benign biliary pathologies (BBP) patients were included in the study. The inclusion criteria for this GBC group were the same as those recruited for tissue samples. The inclusion criteria for the BBP group required patients to be over 18 years of age and to be clinically confirmed to have GD or cholecystitis. The exclusion criteria for both sample groups were patients who were diagnosed with any additional hepatopancreatobiliary diseases. Blood samples were collected in 10 ml EDTA vials and processed within 6 h of collection. Processing included separation into plasma by allowing the tube to stand erect. The plasma was then carefully transferred to a fresh 15 ml falcon tube and centrifuged at 3000 rpm for 30 min to remove any debris. Thereafter, the plasma was aliquoted and stored at − 80 °C until required. The TNM staging and historical gallstone status for the GBC patients were recorded. There were missing data for 23 patients (38.89%) and 14 patients (25.9%) for TNM staging and historical gallstone disease status, respectively.

All demographic and clinical information was captured for each patient in REDCap (V.11.3.4, Vanderbilt University).

### Tissue homogenisation and protein extraction

Between 15 and 20 mg of the tissue was resuspended in a 500 µl ATL Lysis Buffer (Qiagen, Hilden, Germany) and homogenised using the Tissue Ruptor (Qiagen, Hilden, Germany) until all the tissue was visibly in solution. The total volume was determined and 4× volume cold acetone (stored at  −20 °C) was added and incubated at − 20 °C for 60 min. Thereafter, the resulting precipitant was centrifuged at > 14,000×*g* for 10 min. The pellet was washed with 100 µl ice-cold ethanol and the pellet dried for approximately 1 min. The pellet was resuspended in 200 µl 2% SDS in 50 mM Tris–HCl pH8 supplemented with PhosSTOP phosphatase inhibitors (Roche, Basel, Switzerland). The solution was then sonicated using probe sonication; 9 cycles of 10 s with 10 s on ice at 70% power. The solution was then centrifuged at > 14,000×*g* for 10 min and the supernatant was transferred to a 0.5 ml Eppendorf tube. The centrifugation was repeated, and the protein was quantified using the 2-D Quant kit (Cytiva, Massachusetts, USA) as per the manufacturer’s instruction.

### Protein aggregated capture (PAC)

Protein aggregated capture (PAC) was performed on all tissue samples (GBC, GD, and normal tissues). Proteins were reduced with 10 mM dithiothreitol (DTT) and incubated for 30 min at 37 °C. Thereafter, the proteins were alkylated with the addition of 20 mM iodoacetamide (IAA) (final concentration from 1 M stock solutions) and incubated for 30 min at room temperature in the dark.

PAC was performed as previously described [29] with modifications (Additional file 8: Table S1 for plate layout). MagReSyn™ Hydroxyl beads (ReSyn Biosciences, Edenvale, South Africa) were used for protein capture. A protein:bead ratio of 1:4 (by weight) was used for PAC; 20 µg of protein was used per sample and trypsin was used in a ratio of 1:10 (protease:protein) for digestion (4 h at 37 °C). Acetonitrile (ACN) (final concentration of 70%) was used for on-bead protein aggregation which was allowed to occur for 10 min without agitation. The PAC protocol, including on-bead digestion, was automated on a KingFisher™ Duo (Thermo Fisher Scientific, Massachusetts, USA) purification system. Once completed, the plate was transferred to a magnetic rack to recover digested peptides. The peptides were transferred to a 0.5 ml protein LoBind tube (Eppendorf, Hamburg, Germany) and recovered volumes were determined. Digestion was terminated by the addition of TFA (trifluoroacetic acid) to a final of 0.5%. The samples were frozen at − 80 °C and dried at − 4 °C using a CentriVap vacuum concentrator (Labconco, Missouri, USA). The peptides were resuspended in 2% ACN and 0.2% Formic Acid and quantified using the Pierce™ Quantitative Colourimetric Peptide Assay (Thermo Fisher Scientific, Massachusetts, USA) as per the manufacturer’s instruction.

### Hydrophilic interaction liquid chromatography (HILIC)

Hydrophilic interaction liquid chromatography (HILIC) was performed on all plasma samples. Proteins were reduced with 10 mM dithiothreitol (DTT) and incubated for 30 min at 37 °C. Thereafter, the proteins were alkylated with the addition of 20 mM iodoacetamide (IAA) and incubated for 30 min at room temperature in the dark. A total of 30 µg of protein was reduced and alkylated and added to the HILIC binding buffer in a 1:1 ratio (200 mM NH_4_Ac, 30% ACN, pH 4.5).

MagReSyn™ HILIC beads (ReSyn Biosciences, Edenvale, South Africa) were used for protein capture, and a protein:bead ratio of 1:4 (by weight) was utilised. The beads were then equilibrated using equilibration buffer (100 mM NH_4_Ac, 15% ACN, pH 4.5), followed by protein binding onto the HILIC beads for 30 min. Thereafter, two washes using 95% ACN were performed. Following the washes, digestion was performed using trypsin and endoproteinase Lys-C (1:20 and 1:100 ratio of protease:protein, respectively). Digestion occurred for 2 h at room temperature. Once digestion was completed, the enzyme digestion was terminated using 1% TFA. The HILIC protocol was performed using an automated KingFisher™ Flex (Thermo Fisher Scientific, Massachusetts, USA) purification system [27]; each step was performed in a fresh 96-deep well plate. Once the digestion was completed and terminated, the peptides were recovered using a magnetic rack and transferred to fresh 0.5 ml protein LoBind tubes (Eppendorf, Hamburg, Germany). The samples were frozen at - 80 °C and then dried at − 4 °C using a CentriVap vacuum concentrator (Labconco, Missouri, USA). The peptides were then resuspended in 2% ACN and 0.2% Formic Acid and quantified using the Pierce™ Quantitative Colourimetric Peptide Assay (Thermo Fisher Scientific, Massachusetts, USA) as per the manufacturer’s instruction.

### High pH reverse phase fractionation

For high pH reverse phase (RP) fractionation, an aliquot of each prepared GBC and GD samples were pooled together in their respective groups (~ 30 µg of each pool used for high pH RP fractionation). A linear gradient of 5 – 45% Solvent B (Solvent A: 20 mM NH_4_OH; Solvent B: 20 mM NH_4_OH/80% ACN) over 10 min at a flow-rate of 75 µl min^−1^ was employed on a Hypersil GOLD C18 column (1 mm × 15 cm, 3 μm particle size) maintained at 50 °C to fractionate the pooled samples; fractions were collected at 30-s intervals between 13 and 23 min. Appropriate fractions were collected and concatenated together (Additional file 1: Fig. S1, Additional file 8: Table S2), concentrated using a CentriVap vacuum concentrator (Labconco, Missouri, USA), and resuspended before LC–MS injection.

### Liquid chromatography-mass spectrometry (LC–MS) data acquisition

Tryptic peptides (~ 500 ng for sequential window acquisition of all theoretical fragment ion spectra (SWATH) analysis of each sample were analysed using a Evosep One LC system (using Evotip C18 trap column loading system) coupled to an AB Sciex 6600 TripleTOF mass spectrometer (AB Sciex, Massachusetts, USA). Peptide samples were separated on an Evosep performance column (8 cm × 150 µm) packed with 1.5 µm Dr Maisch C18 beads. The column was maintained at 35 °C using the 60SPD method. The peptides were then eluted over 21 min with a gradient of 0–35% Solvent B (Solvent A: 0.1% Formic Acid; Solvent B: 100% ACN/0.1% Formic Acid).

For data-dependent (concatenated fractions) acquisition (DDA), ~ 500 ng of tryptic peptides of each sample were analysed using a Dionex Ultimate 3000 RSLC system coupled to an AB Sciex 6600 Triple TOF mass spectrometer. Peptide samples were inline desalted using an Acclaim PepMap C18 trap column (75 μm × 2 cm; 2 min at 5 μl min^−1^ using 2% ACN/0.2% FA). Trapped peptides were gradient eluted and separated on a Waters Acquity CSH C18 NanoEase column (75 μm × 25 cm, 1.7 μm particle size) maintained at 45 °C at a flow-rate of 0.3 μl min^−1^ with a linear gradient of 4 – 40% Solvent B over 45 min (Solution A: 0.1% Formic Acid; Solvent B: 80% ACN/0.1% Formic Acid). Precursor (MS) scans were acquired from *m/z* 400–1500 (2^+^–5^+^ charge states) using an accumulation time of 200 ms followed by 40 fragment ion (MS/MS) scans, acquired from *m/z* 100–1800 with 20 ms accumulation time each. For SWATH, precursor scans ranged from *m/z* 400–900 using an accumulation time of 100 ms, and fragment ions were acquired from *m/z* 100–1800 with 15 ms accumulation time per window across 60 variable-width windows that overlapped by 0.5 Da.

### LC–MS data processing

A spectral library was built in Spectronaut v16 (Biognosys Schlieren, Switzerland) using the Pulsar search algorithm. Specific trypsin digestion was used for the enzyme setting. A peptide length of 7–52 was used and 2 missed cleavages per peptide were allowed. Carbamidomethylation was added as a fixed modification, N-terminal acetylation and methionine oxidation were added as variable modifications. A Swissprot Human FASTA file (downloaded on 12 June 2021) including common contaminating proteins was used as the search database. For DIA analysis, the standard identification and quantification settings were used for data processing except for data filtering which was set at q-value percentile (0.5 fraction) without imputation (i.e., precursors need to be identified in at least 50% of runs to be included in the analysis). A q-value ≤ 0.05 cut-off was applied at the precursor peptide and protein levels. Quantification was performed at the MS2 level. Label-free cross-run normalization was employed using a global normalization strategy.

### Retrospective power analysis

To determine the appropriate fold-change cut-off, a retrospective power analysis was performed using the MSstats package (Northeastern University, MSstats 4.4.1 (Bioconductor version: Release 3.15, R v4.2.0). The dataProcess function was performed first to normalise the output data from Spectronaut (fragment level peak area of all identified proteins). Thereafter, the groupComparison function was performed to compare the protein changes between GBC, GD, normal, and BBP groups. Finally, the designSampleSize function was applied; this function determines the minimum number of replicates required to achieve a desired statistical power. The parameters were FDR = 0.05, and n = minimum sample size for each comparison (5 for GBC vs normal, 13 for GBC vs GD and 54 for GBC vs BBP plasma). At power = 0.8, proteins that show a fold-change ≥ 5.2, ≥ 2.775, and ≥ 1.6 are significantly dysregulated for the GBC vs Normal, GBC vs GD, and GBC vs BBP plasma comparisons, respectively.

### Pathway and network analysis

Pathway analysis was performed on all the significantly dysregulated proteins identified to determine enriched pathways. REACTOME (v3.7) [30] was used for pathway analysis, and the top 10 significantly (p < 0.05) enriched pathways were selected. Network analysis and visualisation were performed using Cytoscape (v3.8.2) [31] and stringAPP (v1.7.0) [32]. The proteins were queried with filters including Species: *Homo sapiens* and zero additional interactors. Within the network, single non-interacting proteins were excluded. The identified dysregulated proteins were also inputted onto the PANTHER™ Classification System (v17.0) to identify the molecular functions of target proteins [33].

### Statistical analysis

The demographic and clinical characteristics were analysed using R (V4.0.2 and R Studio v1.4.1717). All data were nonparametric and a p < 0.05 was considered significant. The categorical and continuous data were analysed using the Fisher’s Exact and Mann–Whitney U Tests, respectively. Using Statistica (v13.5) a Kruskal–Wallis ANOVA by Ranks with post-hoc analysis was performed to determine any associations between dysregulated protein expression and sex age range. An unsupervised principal component analysis (PCA) was performed using PAST (V4.07b) on the commonly dysregulated proteins between tissue and plasma in GBC patients. For the PCA, a correlation matrix was used to explain the maximal variance in samples that would permit delineation of the disease contexts. Significant loadings for PCA analysis were determined using the following equation:

$$\frac{1}{\sqrt{n\left(variables\right)-1}}.$$

**Fig. 1:**
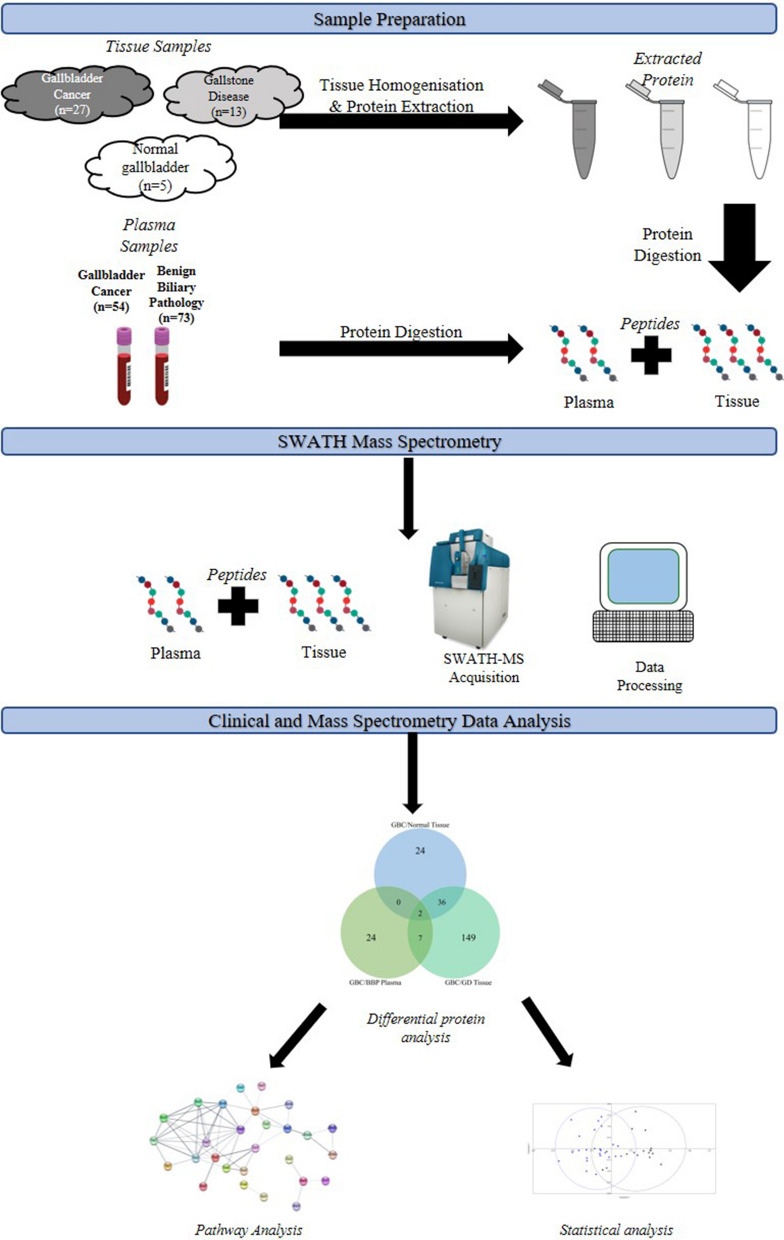
Flow chart of the summary of methods used. SWATH-MS analysis was used to profile biosamples from patients with gallbladder cancer and benign biliary pathologies, such as gallstone disease. Proteins dysregulated in gallbladder cancer patients were identified. Subsequently, pathway and statistical analyses were conducted

The values generated by the equation were used to determine the positive and negative significant loadings for PCA analysis. Thereafter, a Spearman’s Rank Correlation test was conducted to determine whether protein expression correlated across the sample types. Moreover, hierarchical clustering analysis was performed on all the differentially expressed proteins for each of the group comparisons. The hierarchical clustering analysis generated on Spectronaut was used for this analysis. A summary of the methods used is shown in a flowchart represented in Fig. 1.

## Results

### Clinical and demographic characteristics of patients

The differences in the routine clinical blood tests performed between the independent patient cohorts are shown in Additional file 8: Tables S3 and S4. Majority of the GBC patients presented with adenocarcinomas. As expected, liver function tests such as total bilirubin, direct bilirubin, alkaline phosphatase (ALP), gamma-glutamyl transcriptase (GGT), and aspartate transaminase (AST) were elevated in gallbladder cancer patients. Furthermore, the clinical inflammatory markers, white cell count (WCC) and C-reactive protein (CRP) were both raised in GBC patients. The TNM staging and GD history for each patient in the GBC plasma cohort are shown in Additional file 8: Table S5. A total of 14 (25.9%) patients had a history of GD, 26 (48.2%) patients had no history of GD, and 14 (25.9%) patients had an unknown history.

### SWATH-MS analysis of tissue and plasma cohorts

The project-specific spectral library was built in Spectronaut v16 using the Pulsar search algorithm. There were 87,341 precursors, 65,725 modified peptides, 62,204 peptides, and 57,435 proteotypic peptides identified in tissue spectral library. The identified peptides matched to 6204 protein groups. In the GBC/Normal comparison, SWATH-MS identified 36,606 peptides matching 2955 proteins groups across all runs; there were 12,527 peptides matching 1834 protein groups in common across 50% of the total runs. For the GBC/GD group, there were 36,830 peptides matching 2951 protein groups across all runs; and there were 11,261 peptides matching 1674 protein groups in common across 50% of the runs. In the GBC/BBP group, 3310 peptides matching 260 protein groups were present in the custom-built spectral library and there were 2577 peptides matching 226 protein groups which were in common across 50% of the total runs. Details of precursors, peptides and protein groups for each study sample is described in supplementary tables (Additional file 8: Tables S6, S7, S8).

There were a total of 62 proteins dysregulated (38 upregulated and 24 downregulated) in the GBC/Normal group (Additional file 8: Table S9), 194 dysregulated proteins (88 upregulated and 106 downregulated) in the GBC/GD group (Additional file 8: Table S10), and 33 dysregulated proteins (12 upregulated and 21 downregulated) in the GBC/BBP group (Additional file 8: Table S11). A sub-analysis was further performed to compare GBC patients with GD history with those without a history of GD and showed two proteins, S100A8 and S100A9, were downregulated in patients with GD history compared to those without GD history.

### Commonly dysregulated proteins between the tissue and plasma cohorts

**Fig. 2:**
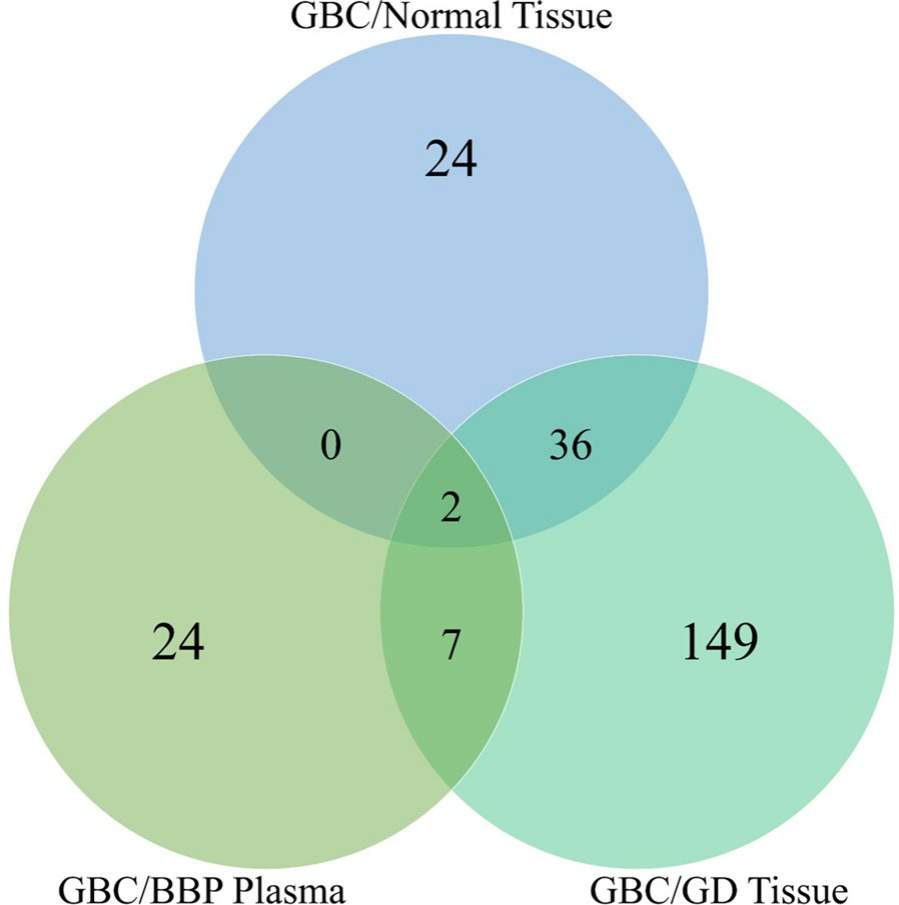
Venn diagram for common dysregulated proteins among sample group comparisons. There is a total of 62, 194, and 33 identified dysregulated proteins across GBC/Normal tissue, GBC/GD tissue, and GBC/BBP plasma groups, respectively. There are commonly dysregulated proteins as indicated by the overlaps. The Venn diagram was generated using Venny (v2.1.0)

Furthermore, we identified proteins that were commonly and uniquely dysregulated across the different patient groups (Fig. 2). There were 24, 149, and 24 dysregulated proteins unique to GBC/Normal, GBC/GD, and GBC/BBP sample groups, respectively. There were 38 dysregulated proteins common to the GBC/Normal and GBC/GD tissue groups. Seven proteins (Apolipoprotein A-1 (APOA1), Apolipoprotein A-2 (APOA2), Retinol-binding protein 4 (RET4), Transthyretin (TTR), Hemopexin (HEMO), Haemoglobin subunit alpha (HBA), Haemoglobin subunit beta (HBB)) were common between GBC/GD and GBC/BBP groups. Also, two proteins were common among all three groups, these are Polymeric immunoglobulin receptor (PIGR) and Apolipoprotein E (APOE) (Additional file 8: Table S12). Henceforth, we will collectively refer to these 9 proteins as “Commonly dysregulated proteins (CDPs)”. These CDPs are dysregulated in the same direction (either upregulated or downregulated) across the sample types (tissue and plasma). Additionally, a subset analysis was conducted on the dysregulated proteins in GBC plasma patients to identify proteins with significant alteration in the different tumour staging. Only one protein was significantly altered; APOE and Inter-alpha-trypsin inhibitor heavy chain H3 (ITIH3) were significantly elevated in non-metastatic (Stage I, II, and III) GBC plasma patients compared to metastatic (Stage IV) (Additional file 8: Table S13). Also, a Kruskal–Wallis H test was performed to determine if age and sex had any effect on protein expression and it was determined that they did not affect the expression of the CDPs. The Log_2_ quantities for the CDPs of each patient are shown in Additional file 2: Fig. S2 and Additional file 3: Fig. S3.

### Pathway and network analyses of differentially expressed proteins

**Fig. 3:**
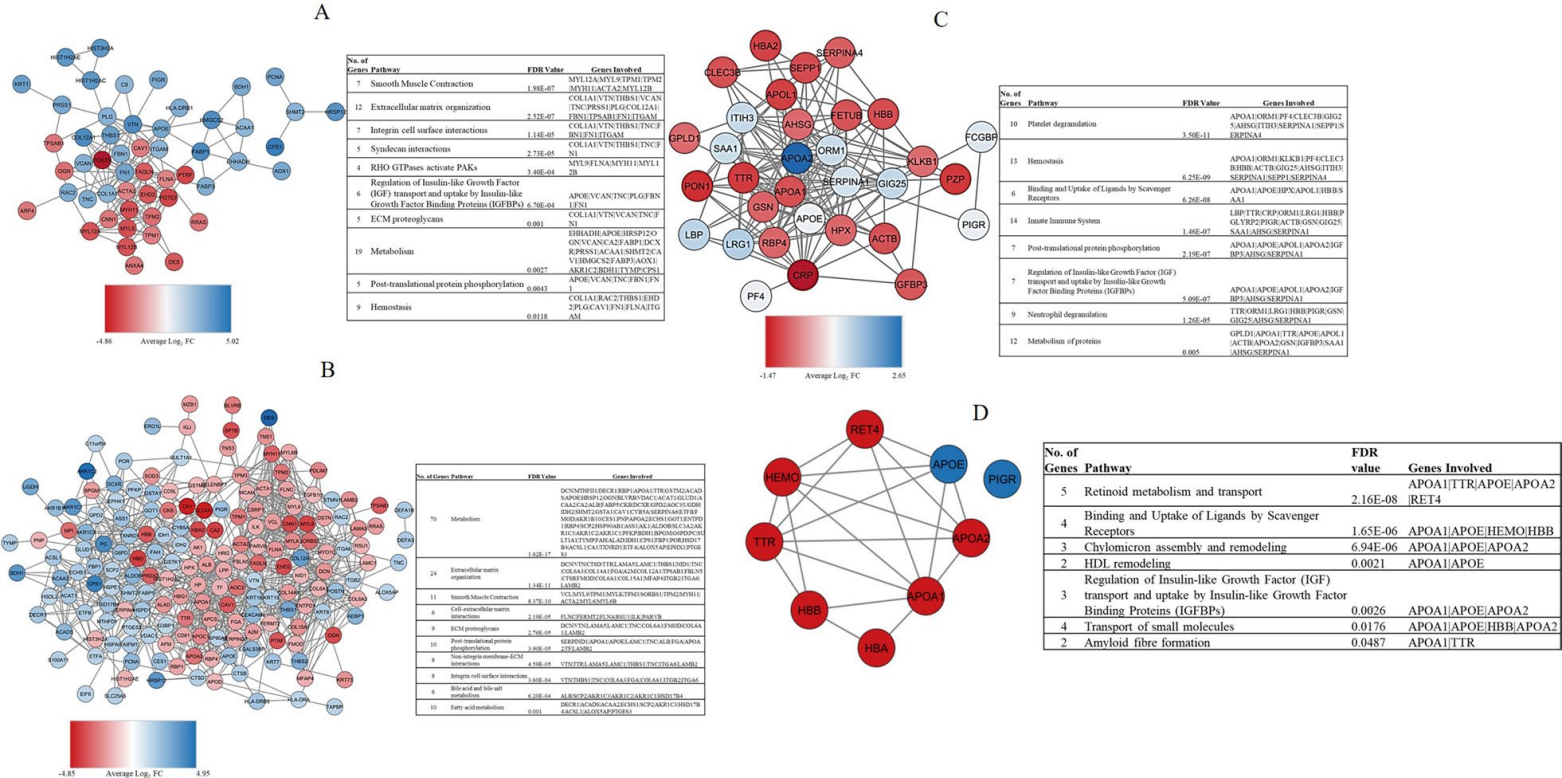
Pathway and network analyses of dysregulated proteins. Network and dysregulated pathways of **A** Gallbladder cancer tissue compared to normal gallbladders. **B** Gallbladder cancer tissues compared to gallstone tissues. **C** Gallbladder cancer plasma compared to benign biliary pathologies plasma samples. The colour change is determined by the average Log_2_ fold change of the protein. **D** Network analysis shows the intricate relationships between the CDPs. Red and blue indicate downregulated and upregulated proteins in GBC across the groups, respectively

Pathway analysis was performed to help identify the molecular pathways in which the dysregulated proteins are involved (Fig. 3). The top downregulated pathway for GBC/Normal is smooth muscle contraction. The top upregulated pathways are integrin cell surface interactions, extracellular matrix organisation, and metabolism. Like the GBC/Normal group, the top downregulated pathways for GBC/GD are smooth muscle contraction, and cell-extracellular matrix interactions. Both the upregulated and downregulated proteins in the GBC/GD group showed enrichment in metabolism and extracellular matrix organisation pathways. For the GBC/BBP group, the dysregulated proteins were shown to be involved in platelet degranulation, haemostasis, and innate immune system pathways. Additionally, network analysis demonstrated that there was a variety of interactions between the CDPs and only PIGR was not involved in the network (Fig. 3D).

To determine pathways that may be unique to GBC, pathway analysis was first performed on the resulting 38 commonly dysregulated proteins from the GBC/Normal and GBC/GD tissue groups (Additional file 4: Fig. S4). The analysis showed the downregulation of the smooth muscle contraction pathway in GBC tissues. Then, another analysis investigating pathways commonly enriched by the proteins (CDPs) found to be dysregulated at both the tissue and plasma level, demonstrated that pathways associated with metabolism were the most enriched. In addition, the gene ontology enrichment analysis for dysregulated proteins for all the groups determined that the molecular functions of most of the proteins are related to binding and catalytic activities (Additional file 5: Fig. S5).

### Hierarchical clustering, principal component analysis, and Spearman’s rank correlation test of the CDPs

Hierarchical clustering was performed on all of the quantified proteins for GBC/Normal, GBC/GD, and GBC/BBP (Additional file 6: Fig. S6). The tissue groups showed distinct clustering by condition (tumour and control), however, the conditions for the plasma dataset showed some overlap.

**Fig. 4:**
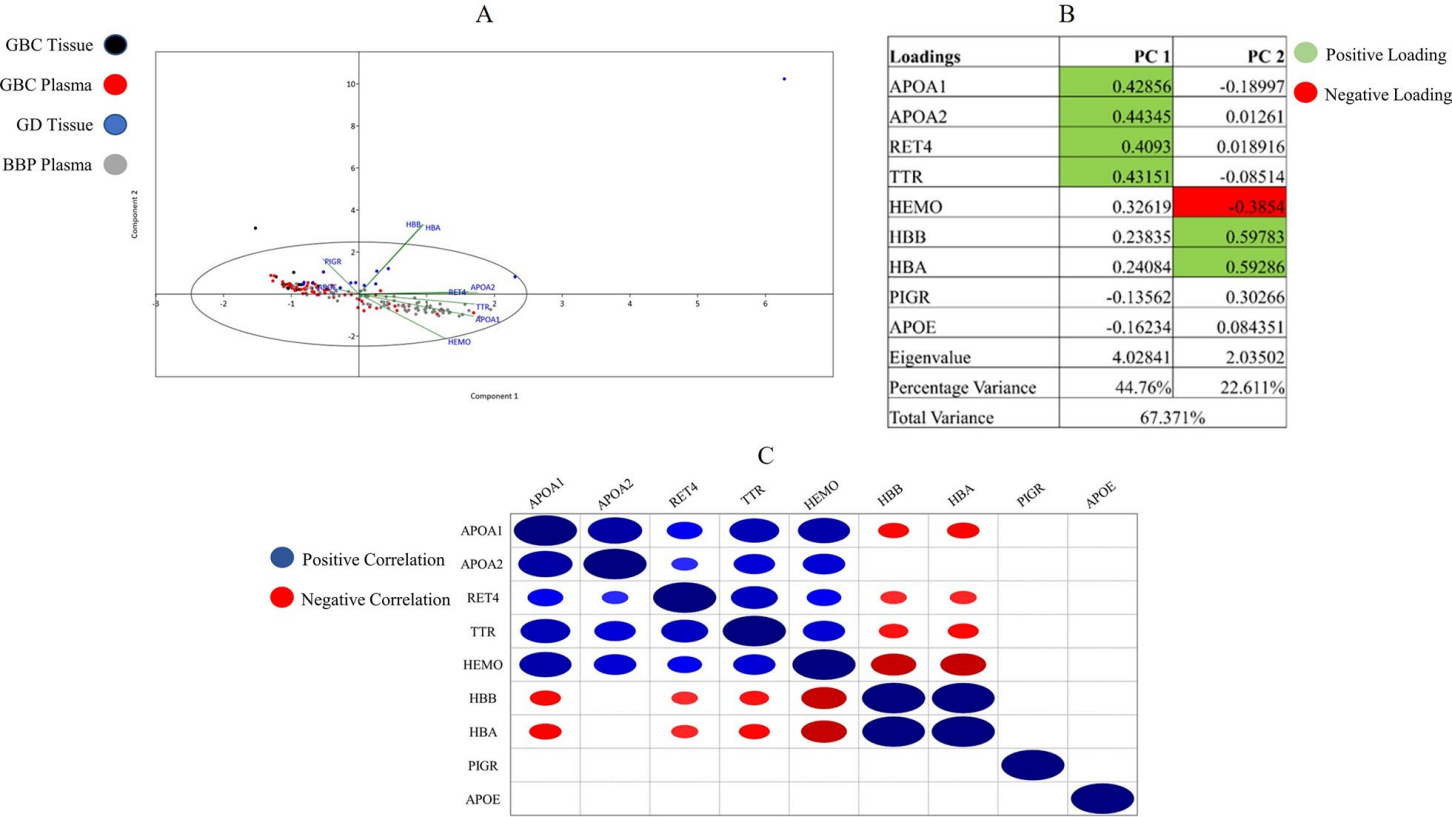
Principal component analysis and Spearman’s Rank Correlation Test for "Commonly dysregulated proteins (CDPs)". **A** The scatterplot indicates PC1 vs PC2 of the CDPs. Black dots indicate GBC tissue patients, red dots indicate GBC plasma patients, blue dots indicate GD patients and grey dots indicate BBP patients. **B** The loadings for the CDPs for each PC. Green highlighted values are significantly positive loadings and red highlighted values are significantly negative loadings. **C** The Spearman’s Correlation Rank Test indicates significant positive (blue dots) and negative (red dots) between the CDPs

Thereafter, to determine the ability of the CDPs to distinguish between GBC and control, and to reduce data dimensionality, principal component analysis (PCA) was performed. Visually, GBC tissue (black) and GBC plasma (red) samples showed considerable overlap following the reduction of dimensionality (Fig. 4A). Specifically, the two principal components (PCs) that were generated accounted for 67.37% of the variation. GBC tissue and plasma clustering was influenced by PC1 (44.76% variation) with strong contributions from APOA1, APOA2, RET4, and TTR. As mentioned previously, there are specific interactions between APOA1, APOA2, RET4, and TTR as indicated by the network analysis (Fig. 3D). Moreover, the relationship between GBC tissue and plasma was delineated from GD and BBP on the vertical axis (PC2; 22.61% variation) by strong positive influences from HBB and HBA and a negative influence from HEMO (Fig. 4B).

The Spearman’s Rank Correlation test was performed to determine the pattern of CDPs expression. Specifically, the correlation between proteins within GBC tissue and plasma samples was identified (Fig. 4B; Additional file 7: Fig. S7). The proteins APOA1, APOA2, RET4, TTR, and HEMO were all significantly positively correlated with each other and are involved in an intricate network; sharing similar pathways such as retinoid metabolism. Strong positive contributions on PC1 by APOA1, APOA2, RET4, and TTR support the significant correlations between these proteins. HBB and HBA are also significantly positively correlated with each other (Fig. 4D). However, HBA and HBB are significantly negatively correlated with APOA1, RET4, TTR, and HEMO. The strong positive contributions on PC2 by HBB and HBA with a negative contribution by HEMO are also supported by the positive correlation between HBB and HBA, and the inverse correlation between HBB/HBA and HEMO (Fig. 4B, C).

Although also downregulated in GBC, HBA and HBB are negatively correlated with other CDPs, such as APOA1, suggesting a negative scale continuum of expression which could be indicative of varying molecular functions. It is noteworthy that HBB and HBA are present at higher abundance compared to the other proteins. Both PIGR and APOE are not significantly correlated with any other proteins.

We conducted an additional correlation test to determine whether the specific levels of CDPs correlated across tissue and plasma. No significant correlations were identified.

## Discussion

Gallbladder cancer (GBC) has a poor prognosis with a growing incidence and mortality worldwide. In most cases, GBC is detected in the advanced stage leading to a poorer prognosis. This calls for a better understanding of the disease progression and the identification of biomarkers. Importantly, the variations observed in both incidence and mortality across different regions reinforce the possible involvement of molecular, clinicopathological and environmental factors. Some published studies have determined the molecular profiles highlighting potential mechanisms of progression and biomarkers for the disease. However, this information has been lacking in African patients. To our knowledge, this is the first study to observe potential molecular mechanisms and markers linked to GBC in a South African cohort by conducting proteomics profiling of tissue and plasma samples.

We found that the most prevalent type of GBC cancer in our sample cohort were adenocarcinomas, corroborating other published findings [10]. GBCs are difficult to diagnose therefore sensitive and specific markers are required. In this study, we determined that CA19-9 is elevated in GBC patients. The tumour marker, CA19-9 has been widely studied for its diagnostic and prognostic utility in GBC [34, 35]. Most liver function tests were significantly elevated in GBC patients compared to patients in the GD or BBP group (Additional file 8: Tables S3 and S4). It is well-known that GBC progression can affect the functioning of the liver, consequently altering the levels of liver parameters such as bilirubin. Bilirubin, which is produced and excreted by the liver via heme degradation, can accumulate due to biliary obstruction causing jaundice [36, 37], a condition which was more prevalent in the GBC groups. GBC patients also showed raised levels of the clinical inflammatory markers, WCC and CRP, suggesting increased inflammation. WCC is regarded as a non-specific inflammatory marker that is often increased in acute or chronic infections [38]. CRP is primarily expressed in hepatocytes and its expression is regulated by interleukin-6 (IL-6), a well-known pro-inflammatory cytokine [39]. An elevated level of CRP is associated with an increased risk of developing GBC [40, 41]. However, these routine clinical blood tests are non-specific for GBC and are observed in several disease conditions including a wide range of malignancies [42].

The present study identified dysregulated proteins in a cohort of South African GBC patients. The top upregulated pathway enriched by these proteins in gallbladder cancer tumours is the extracellular matrix (ECM) organization pathway. The ECM pathway regulates several key hallmarks of cancer such as proliferation, evasion of the immune response, and cell death and therefore is crucial in promoting tumour progression and metastasis [27, 43, 44]. Due to its biological functions, the upregulation of components of the ECM organization pathways may be crucially involved in the pathogenesis of GBC. The top downregulated pathway identified in GBC tumours is smooth muscle contraction. This process in the gallbladder is regulated by the hormone cholecystokinin (CCK) which induces gallbladder contraction by its membrane receptor (CCKR) [45–47]. A pilot study looked at the expression of CCKRs in normal gallbladder tissues, gallstones, and gallbladder tumours and observed a decrease in the expression of CCKRs in the tumour samples, although this was not significant [48]. Oxidative stress resulting from dysfunction in gallbladder contraction can damage CCKRs and lead to altered lipid metabolism and induce inflammation [49–51]. In the present study, we also found an elevation of clinical inflammatory markers and dysregulated lipid metabolism (Additional file 8: Table S3; Fig. 3), which may suggest damage to the CCKRs. It is important to note that the downregulation of the smooth muscle contraction pathway may be due to the microenvironment of GBC tumours which consists of predominantly stroma and epithelial cells, compared to normal tissues consisting of muscularis and epithelial cells [52, 53].

The group of proteins referred to as commonly dysregulated proteins (CDPs) in this study, were found to be similarly dysregulated in GBC tissue and plasma (Additional file 8: Table S12). This similarity may suggest that the proteins could be involved in tumour progression and subsequently secreted into the bloodstream [54]. This suggestion is further reinforced by their shared biological pathways (Fig. 3D). The most significant pathway involving the CDPs is retinoid metabolism and transport. Retinoids regulate various cellular processes such as proliferation, differentiation, apoptosis and immunity [55]. The involvement of the downregulated CDPs in this pathway may indicate a reduction of retinoid transport and subsequent anti-tumour functions. The pathway involved in scavenging heme from plasma is also downregulated. Plasma heme originates from the destruction of red blood cells and can undergo autooxidation inducing inflammation and resulting in severe cellular damage. A reduction in the scavenging of heme from plasma may consequently drive the tumourigenic process by maintaining an inflammatory environment [56].

The hierarchical clustering of all the quantified proteins in the tissue and plasma groups was performed. The tissue analysis showed that the quantified proteins were able to separate and cluster the patients distinctly by condition. However, the plasma analysis showed some overlap amongst the GBC and BBP patients. This overlap may be due to various reasons; one possible explanation is that plasma proteins are often expressed ubiquitously in individuals irrespective of diseased state [57]. Another factor is the spectrum of phenotype for GBC patients as some early-stage GBC patients may present with inflammatory disease (BBP) clinically, contributing to the overlap of patient clustering [11, 58].

Interestingly, although APOA1 and HBB/HBA are both downregulated across sample types, there is a significant inverse correlation between these proteins. This is supported by the PCA analysis whereby they make significant contributions to PC1 (APOA1) and PC2 (HBB/HBA) (Fig. 4). This inverse correlation and relationship may indicate the complexity of biological functions even within similar pathways. The main functions of APOA1 and HBB/HBA are cholesterol transport and oxygen transport, respectively. Elevated cholesterol transport in the blood leads to reduced blood oxygen levels. Importantly, a decrease in haemoglobin was observed in GBC patients (Additional file 8: Tables S3 and S4) and increased cholesterol elevates the risk of gallbladder cancer [59]. Their opposite functions may explain their negative correlations [60]. However, a study indicated that significant dysregulations of proteins such as HBB and HBA may be due to erythrocyte contamination in plasma samples [61]. In our study, expression of both HBB and HBA in plasma is also observed in tumours which may suggest that it may be linked to the disease (Additional file 8: Table S12). Gallstone disease is well documented to increase the risk of gallbladder cancer; however not all GBC patients have a history of GD. This study determined that the levels of S100A8 and S100A9 were downregulated in GBC patients with a history of GD compared to those without. These proteins have been documented to be expressed by neutrophils and monocytes as calcium ion sensors [62]. While they are expressed in high levels during inflammation, it was also been demonstrated that at lower levels they promote tumour progression [63]. In a study of various cancer cell lines, it was observed that reduced S100A8/9 levels induced tumour cellular growth and enhances proliferation [62, 63]. GD is considered a precursor for GBC onset which would support the reduced S100A8/9 levels in GD history patients versus no GD history patients [9, 10].

We further performed an analysis to identify significant differences in GBC plasma proteins between non-metastatic and metastatic disease. Of the 33 dysregulated proteins in GBC plasma, APOE and ITIH3 were found to be significantly elevated in non-metastatic compared to the metastatic patients. APOE is a protein involved in cholesterol homeostasis, lipid metabolism and immune suppression [59, 64]. An elevated level of APOE in the blood of non-small cell lung carcinoma (NSCLC) patients was associated with tumour metastases and poor prognosis [65]. Another study demonstrated the overexpression of APOE in stage II colorectal tumours showing it as an independent prognostic factor for overall survival. Taken together, the upregulation of APOE in non-metastatic GBC may suggest its role in promoting tumourigenesis [66]. ITIH3 covalently links to hyaluronic acid, a major component of the ECM. It has been demonstrated to increase cellular attachment in vitro and reduce metastasis in a murine model [67], suggesting its anti-metastatic role.

In this study, we have demonstrated the proteomic signatures in a cohort of GBC patients of African ancestry. GBC has been studied in other populations including Asian populations such as the Chinese and Indian, with observed similarities across populations. For example, previous studies have identified CYFRA 21-1 (soluble fragment of cytokeratin 19) [68] and thymidine phosphorylase (TYMP) [69], to be diagnostic and predictive biomarkers of GBC, respectively. The current study also demonstrated that CYFRA 21-1 and TYMP were significantly dysregulated in GBC patients corroborating their potential utility as biomarkers [70]. In a recent study conducted in India, the authors identified 86 proteins from plasma-derived extracellular vesicles secreted by tumour cells. These extracellular vesicles contain mRNAs, miRNAs, and tumour-associated proteins [17]. Of the 86 proteins identified to be dysregulated in GBC, 15 of those proteins were identified in our patient cohort. These proteins included FLNA, PARVB, PIGR, and RAC2, among others. These aforementioned proteins were found to be dysregulated across both tissue datasets (GBC/Normal and GBC/GD groups) in the present study. Furthermore, PIGR was commonly dysregulated across all datasets and thus expressed in both tissues and blood. While we found some similarities in dysregulated proteins in our cohort compared to other populations, their expression patterns differed. For instance, in a Chinese cohort study using MALDI-TOF MS two proteins; Annexin A4 (ANXA4) and heat shock protein 90-beta (Hsp90β) were similarly dysregulated in our patient cohort [71]. However, ANXA4 was found to be upregulated and Hsp90β was found to be downregulated in the Chinese cohort whereas the inverse was the case in our study.

## Conclusion

This study has demonstrated significantly dysregulated proteins in GBC patients using SWATH-MS. This is the first study to utilise such an approach to identify proteins in patients of African ancestry, providing much-needed molecular data on this group of patients. Importantly, we showed that a subset of these proteins was shown to be expressed similarly in both tissues and plasma samples from independent cohorts. Potentially, these similar patterns of expression observed, reinforce their significance in a GBC context and their utility as potential biomarkers for the disease. However, their expressions would need to be verified in a larger independent cohort and using alternative methods. Furthermore, the involvement of dysregulated proteins in pathways such as smooth muscle contraction and metabolism can help delineate the molecular mechanisms that may be associated with GBC in the patient cohort.

## Study limitations

The main limitation of this study was the low number of patient samples, especially in the normal gallbladder tissue group, and some missing clinicopathological information such as tumour differentiation and carcinoembryonic antigen levels. Additionally, the use of unmatched tissue and plasma samples may have limited the identification of more potential markers of GBC in this patient cohort. Finally, the absence of healthy control plasma samples limits the evaluation of the key identified proteins in healthy individuals. However, future studies will aim to include these samples in the validation cohort.

## Supplementary Information

**Additional file 1: Figure S1.** Elution Profile for High RP Fractionation. Fractionation profile of eluted peptides using a gradient of 20 mM NH_4_OH and 20 mM NH_4_OH/80% acetonitrile using a Hypersil GOLD C18 column (1 mm × 15 cm, 3 μm particle size) maintained at 50 °C over approximately 15 min. Fractions were collected at 30-s intervals between 13–23 min.

**Additional file 2: Figure S2.** Log_2_ Quantities for the CDPs identified in GBC/GD and GBC/BBP comparisons per patient. (A) The log_2_ quantities per patient for the GBC/GD comparison for each CDP. (B) The log_2_ quantities for each patient for the GBC/BBP plasma comparison.

**Additional file 3: Figure S3.** The Log_2_ Quantities for the CDPs identified in GBC/Normal, GBC/GD, and GBC/BBP Comparisons. (A) The individual patient log_2_ quantities for PIGR in GBC/Normal, GBC/GD, and GBC/BBP comparisons. (B) The individual patient log_2_ quantities for APOE in GBC/Normal, GBC/GD, and GBC/BBP comparisons.

**Additional file 4: Figure S4.** Pathway and Network Analyses for the Commonly Dysregulated Proteins between GBC/Normal and GBC/GD tissue groups. Red indicates downregulated proteins, blue indicates upregulated proteins, and grey indicates upregulated in GBC/Normal but downregulated in GBC/GD.

**Additional file 5: Figure S5.** Annotated molecular functions for Dysregulated Proteins Identified. Pie charts representing the molecular functions of dysregulated proteins in (A) GBC tumours compared to normal tissues. (B) GBC tumours compared to GD tissues (C) GBC compared to BBP plasma samples. The annotation was conducted using PANTHER v17.0.

**Additional file 6: Figure S6.** Hierarchical cluster analysis for the differentially expressed proteins. Hierarchical cluster analyses are shown in the heatmap and dendrograms for all quantified proteins in the GBC/Normal (A), GBC/GD (B), and GBC/BBP plasma (C) comparisons. The cluster brackets on the right side of the heatmaps indicate proteins clustered together based on detection intensity. The clustering brackets on the top indicate clustering based on similarity across the individual samples. The larger brackets indicate a low similarity and the small brackets indicate a close similarity. Blue to yellow colouring indicates low to high expression of the proteins. The heatmaps were generated in Spectronaut v16.

**Additional file 7: Figure S7.** Spearman’s Rho Values and p-values for correlation of CDPs. The Rho correlation values and the corresponding p-values for the CDPs.

**Additional file 8: Table S1.** 96-Deep Well Plate Setup. **Table S2.** Concatenation of Fractions from High pH RP Fractionation. **Table S3.** Clinical and Demographic Characteristics of Gallbladder Cancer and Gallstone Disease Patients. **Table S4.** Clinical and Demographic Characteristics of Gallbladder Cancer and Benign Biliary Pathology Plasma Patients. **Table S5.** TNM Staging and Gallstone Disease History for GBC Plasma Patients. **Table S6.** The Total Precursors, Peptides, and Protein Groups Identified for Each Individual Patient for The GBC Versus Normal Comparison. **Table S7.** The Total Precursors, Peptides, and Protein Groups Identified for Each Individual Patient for The GBC Versus GD Comparison. **Table S8.** The Total Precursors, Peptides, and Protein Groups Identified for Each Individual Patient for The GBC Versus BBP Plasma Comparison. **Table S9.** All Identified Dysregulated Proteins in GBC Compared to Normal. **Table S10.** All Identified Dysregulated Proteins in GBC Compared to GD. **Table S11.** All Identified Dysregulated Proteins in GBC Compared to BBP Plasma. **Table S12.** Common Dysregulated Proteins Across Group Comparisons with Relative Average Log_2_ Fold Change. **Table S13.** Non-Metastatic vs Metastatic Patients for GBC Plasma Dysregulated Proteins.

## Data Availability

The datasets generated during the current study are available in the repository ProteomeXchange Consortium [72] with the dataset identifier PXD029877.

## Abbreviations

ACN: Acetonitrile
ALBU: Albumin
ALP: Alkaline phosphatase
ANOVA: Analysis of variance
ANXA4: Annexin A4
APOA1: Apolipoprotein 1
APOA2: Apolipoprotein 2
APOE: Apolipoprotein E
AST: Aspartate transaminase
BBP: Benign biliary pathology
CA19-9: Carbohydrate antigen 19-9
CCK: Cholecystokinin
CCKR: Cholecystokinin receptor
CDPs: Commonly dysregulated proteins
CHBAH: Chris Hani Baragwanath Academic Hospital
CRP: C-Reactive protein
CYFRA 21-1: Cytokeratin 19 fragment 21-1
DDA: Data-dependent acquisition
DIA: Data-independent acquisition
DTT: Dithiothreitol
ECM: Extracellular matrix
EDTA: Ethylenediaminetetraacetic acid
FA: Formic acid
FC: Fold change
FINC: Fibronectin
FRIL: Ferritin light chain
GBC: Gallbladder cancer
GD: Gallstone disease
GGT: Gamma-glutamyl transcriptase
Hb: Haemoglobin
HBA: Haemoglobin subunit alpha
HBB: Haemoglobin subunit beta
HEMO: Hemopexin
HILIC: Hydrophilic interaction liquid chromatography
Hsp90β: Heat shock protein 90-beta
IAA: Iodoacetamide
IL-6: Interleukin-6
ITIH3: Inter-alpha-trypsin inhibitor heavy chain H3
LC: Liquid chromatography
LC–MS: Liquid chromatography–mass spectrometry
LFT: Liver function test
Lys-C: Endoproteinase Lys-C
MALDI-TOF MS: Matrix assisted laser desorption ionization-time of flight mass spectrometry
miRNA: MicroRNA
mRNA: Messenger RNA
MS: Mass spectrometry
NH_4_Ac: Ammonium acetate
NH_4_OH: Ammonium hydroxide
NSCLC: Non-small cell lung carcinoma
PAC: Protein aggregated capture
PC: Principal component
PCA: Principal component analysis
PIGR: Polymeric immunoglobulin receptor
RET4: Retinol-binding protein 4
RP: Reverse phase
RPM: Revolution per minute
SDS: Sodium dodecyl sulphate
SWATH-MS: Sequential window of all theoretical fragment ion spectra-mass spectrometry
TFA: Trifluoroacetic acid
TRIS–HCL: Tris hydrochloride
TTR: Transthyretin
TYMP: Thymidine phosphorylase
WCC: White cell count
