# Supplementary figures and images for: Proteomic analysis identifies dysregulated proteins and associated molecular pathways in a cohort of gallbladder cancer patients of African ancestry
Source: Clin Proteomics. 2023 Mar 1;20:8. doi: 10.1186/s12014-023-09399-9 (PMC9976386; doi:10.1186/s12014-023-09399-9)

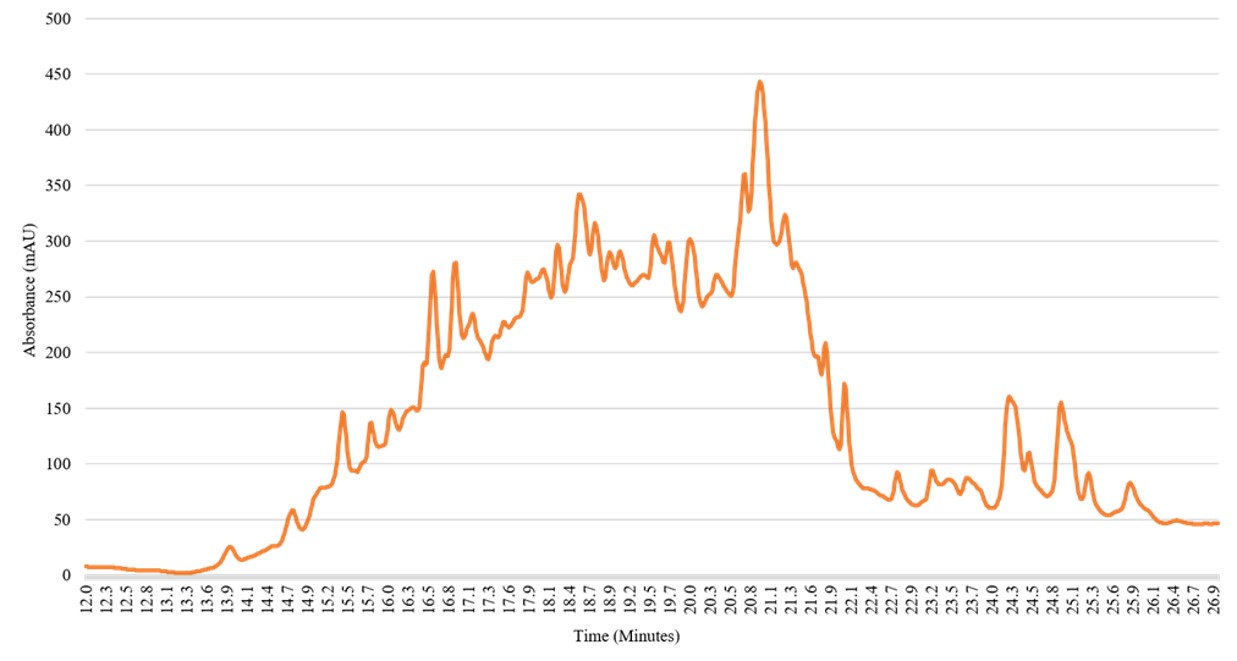

Supplement: Supplementary file 1 — Additional file 1: Figure S1. Elution Profile for High RP Fractionation. Fractionation profile of eluted peptides using a gradient of 20 mM NH4OH and 20 mM NH4OH/80% acetonitrile using a Hypersil GOLD C18 column (1 mm × 15 cm, 3 μm particle size) maintained at 50 °C over approximately 15 min. Fractions were collected at 30-s intervals between 13–23 min. [file 12014_2023_9399_MOESM1_ESM.jpg]

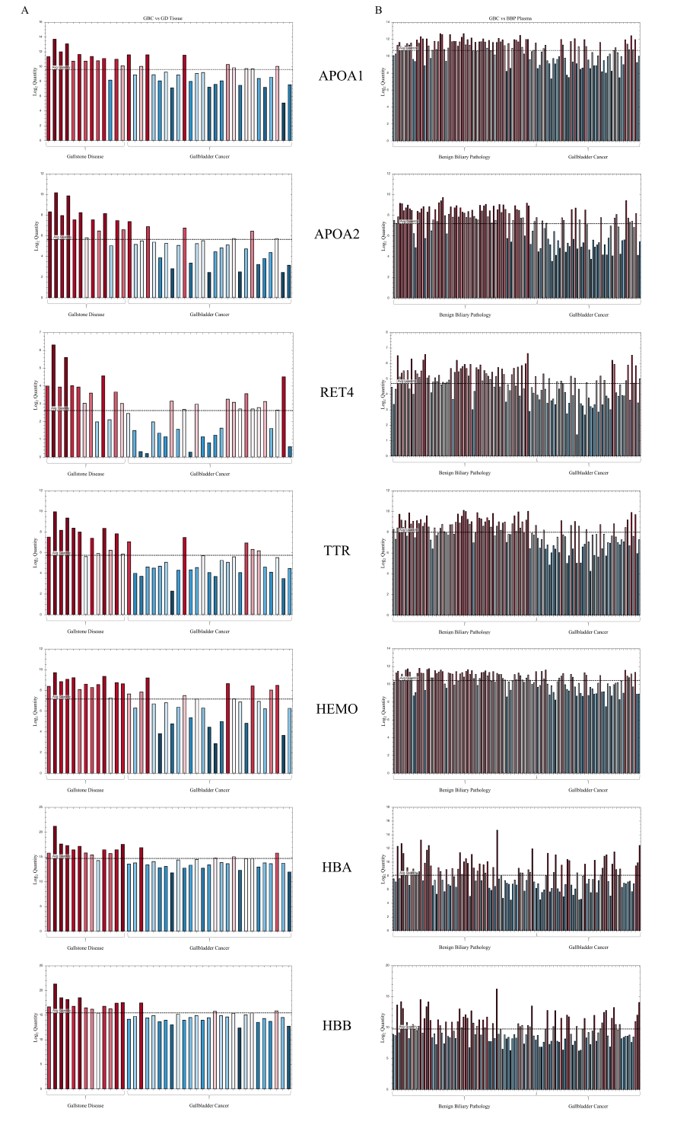

Supplement: Supplementary file 2 — Additional file 2: Figure S2. Log2 Quantities for the CDPs identified in GBC/GD and GBC/BBP comparisons per patient. (A) The log2 quantities per patient for the GBC/GD comparison for each CDP. (B) The log2 quantities for each patient for the GBC/BBP plasma comparison. [file 12014_2023_9399_MOESM2_ESM.jpg]

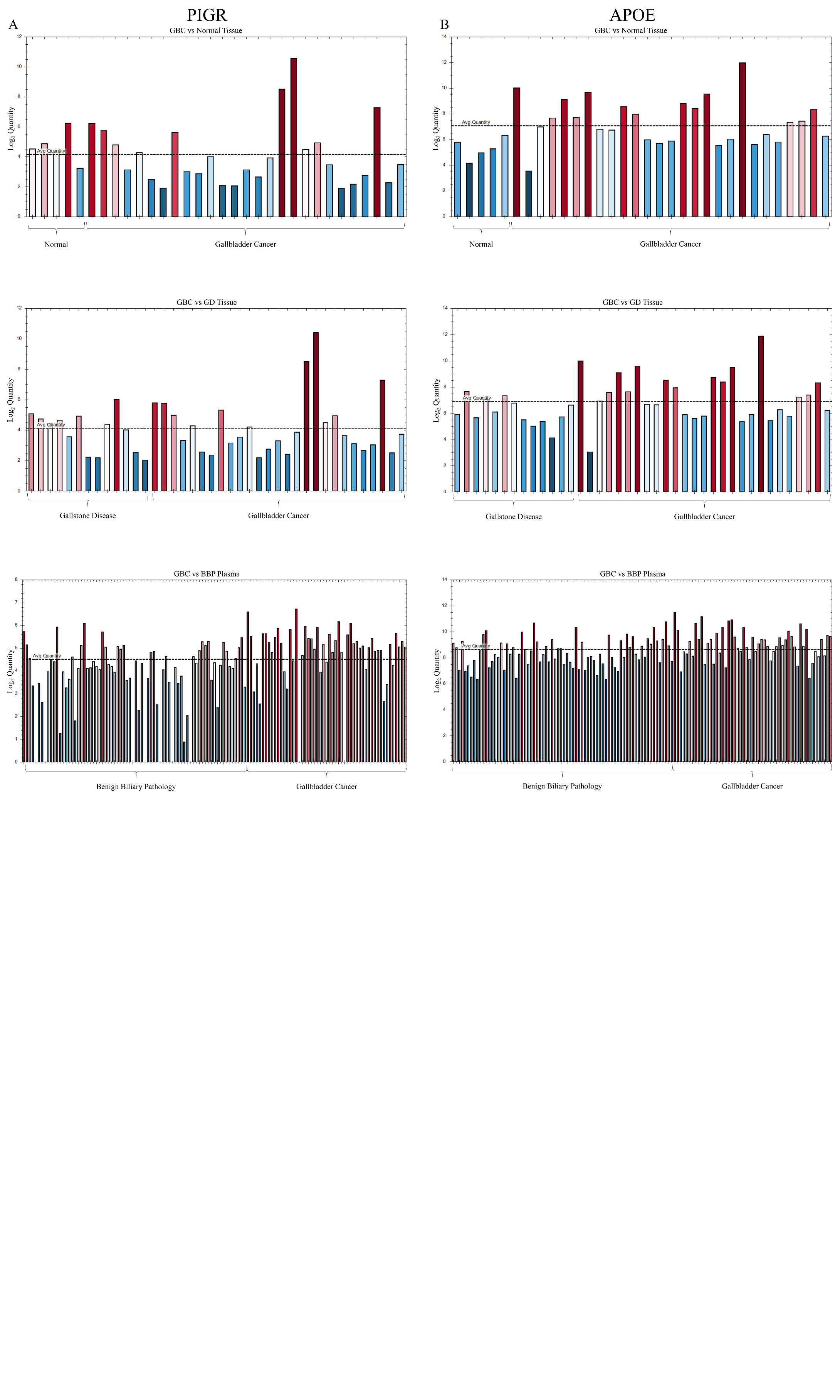

Supplement: Supplementary file 3 — Additional file 3: Figure S3. The Log2 Quantities for the CDPs identified in GBC/Normal, GBC/GD, and GBC/BBP Comparisons. (A) The individual patient log2 quantities for PIGR in GBC/Normal, GBC/GD, and GBC/BBP comparisons. (B) The individual patient log2 quantities for APOE in GBC/Normal, GBC/GD, and GBC/BBP comparisons. [file 12014_2023_9399_MOESM3_ESM.tif]

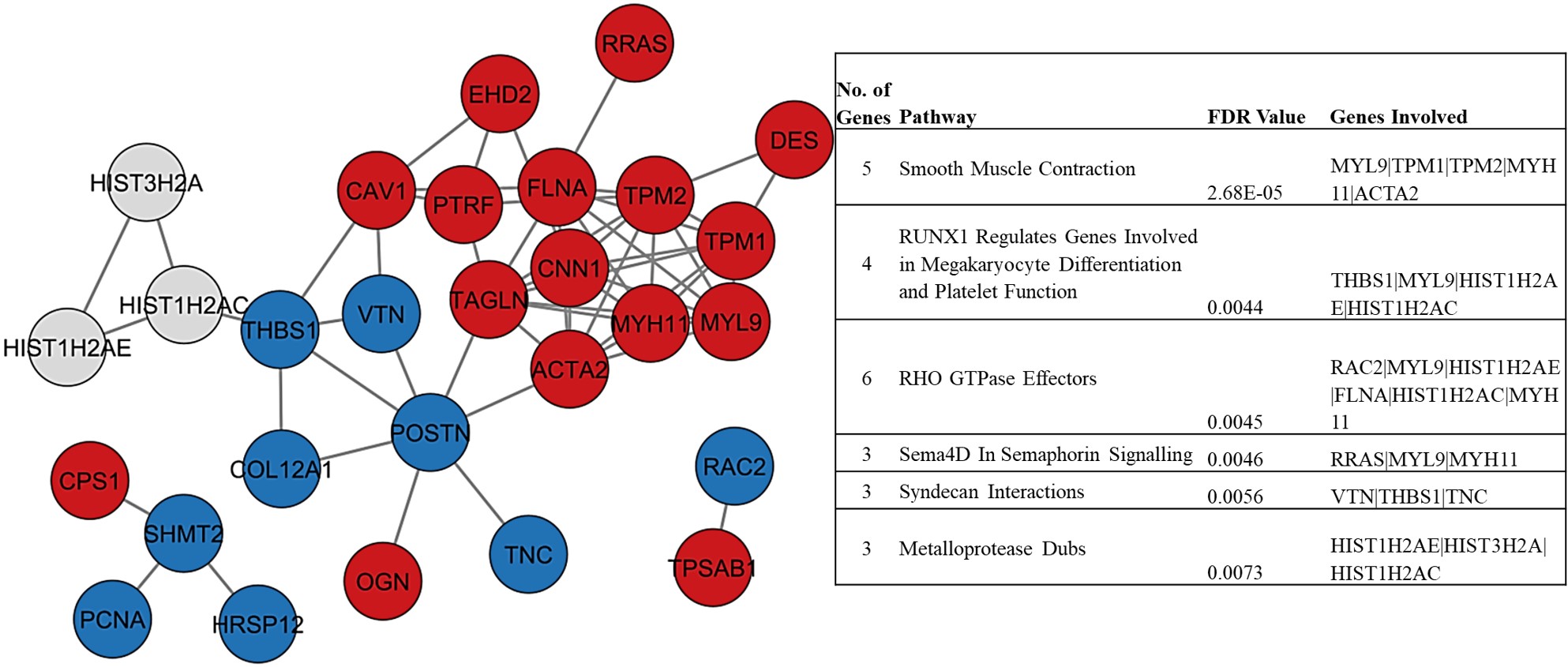

Supplement: Supplementary file 4 — Additional file 4: Figure S4. Pathway and Network Analyses for the Commonly Dysregulated Proteins between GBC/Normal and GBC/GD tissue groups. Red indicates downregulated proteins, blue indicates upregulated proteins, and grey indicates upregulated in GBC/Normal but downregulated in GBC/GD. [file 12014_2023_9399_MOESM4_ESM.jpg]

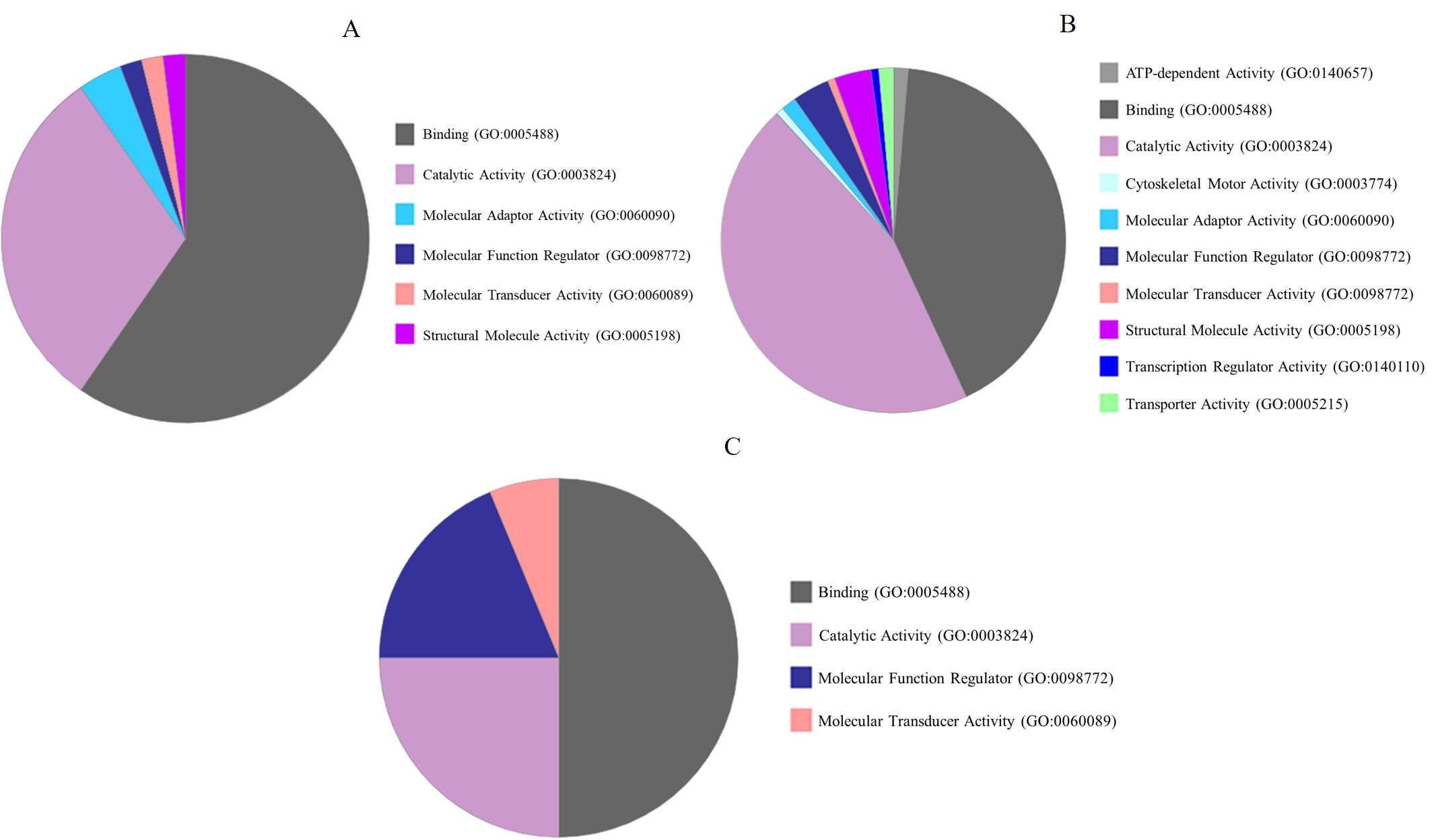

Supplement: Supplementary file 5 — Additional file 5: Figure S5. Annotated molecular functions for Dysregulated Proteins Identified. Pie charts representing the molecular functions of dysregulated proteins in (A) GBC tumours compared to normal tissues. (B) GBC tumours compared to GD tissues (C) GBC compared to BBP plasma samples. The annotation was conducted using PANTHER v17.0. [file 12014_2023_9399_MOESM5_ESM.jpg]

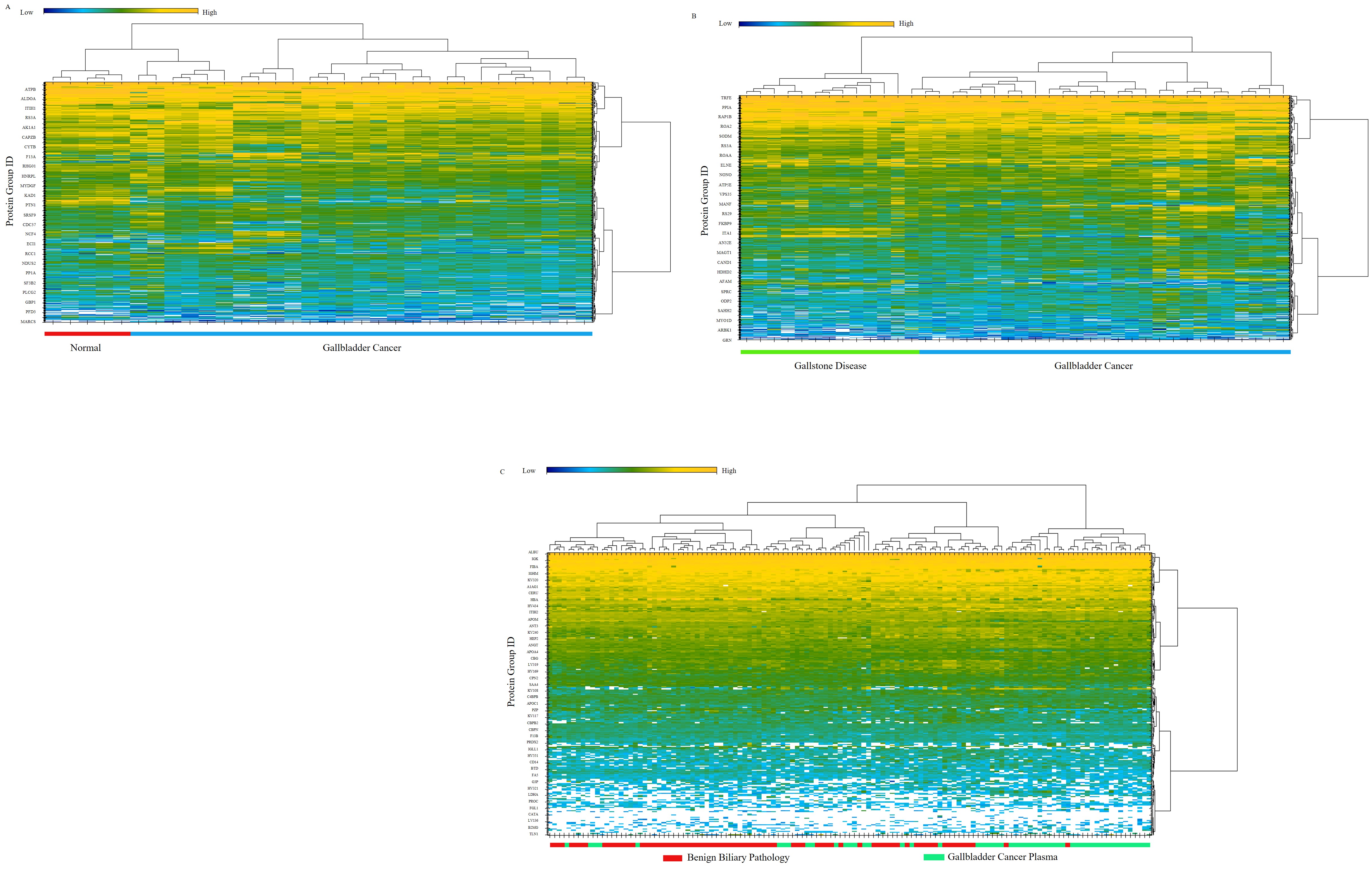

Supplement: Supplementary file 6 — Additional file 6: Figure S6. Hierarchical cluster analysis for the differentially expressed proteins. Hierarchical cluster analyses are shown in the heatmap and dendrograms for all quantified proteins in the GBC/Normal (A), GBC/GD (B), and GBC/BBP plasma (C) comparisons. The cluster brackets on the right side of the heatmaps indicate proteins clustered together based on detection intensity. The clustering brackets on the top indicate clustering based on similarity across the individual samples. The larger brackets indicate a low similarity and the small brackets indicate a close similarity. Blue to yellow colouring indicates low to high expression of the proteins. The heatmaps were generated in Spectronaut v16. [file 12014_2023_9399_MOESM6_ESM.jpg]

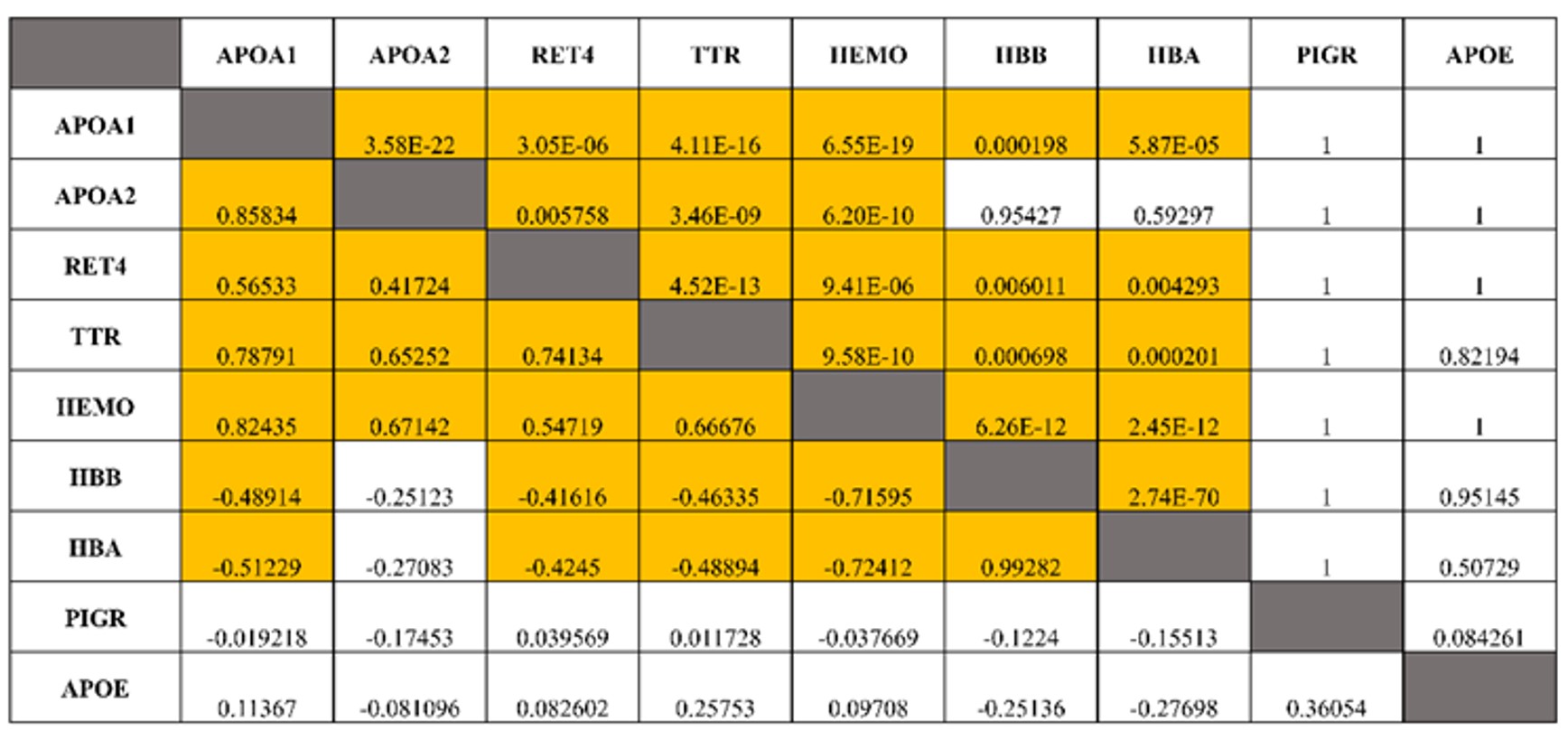

Supplement: Supplementary file 7 — Additional file 7: Figure S7. Spearman’s Rho Values and p-values for correlation of CDPs. The Rho correlation values and the corresponding p-values for the CDPs. [file 12014_2023_9399_MOESM7_ESM.jpg]
