# Supplementary material for: Proteomic analysis identifies dysregulated proteins and associated molecular pathways in a cohort of gallbladder cancer patients of African ancestry
Source: Clin Proteomics. 2023 Mar 1;20:8. doi: 10.1186/s12014-023-09399-9 (PMC9976386; doi:10.1186/s12014-023-09399-9)
Supplement: Supplementary file 8 — Additional file 8: Table S1. 96-Deep Well Plate Setup. Table S2. Concatenation of Fractions from High pH RP Fractionation. Table S3. Clinical and Demographic Characteristics of Gallbladder Cancer and Gallstone Disease Patients. Table S4. Clinical and Demographic Characteristics of Gallbladder Cancer and Benign Biliary Pathology Plasma Patients. Table S5. TNM Staging and Gallstone Disease History for GBC Plasma Patients. Table S6. The Total Precursors, Peptides, and Protein Groups Identified for Each Individual Patient for The GBC Versus Normal Comparison. Table S7. The Total Precursors, Peptides, and Protein Groups Identified for Each Individual Patient for The GBC Versus GD Comparison. Table S8. The Total Precursors, Peptides, and Protein Groups Identified for Each Individual Patient for The GBC Versus BBP Plasma Comparison. Table S9. All Identified Dysregulated Proteins in GBC Compared to Normal. Table S10. All Identified Dysregulated Proteins in GBC Compared to GD. Table S11. All Identified Dysregulated Proteins in GBC Compared to BBP Plasma. Table S12. Common Dysregulated Proteins Across Group Comparisons with Relative Average Log2 Fold Change. Table S13. Non-Metastatic vs Metastatic Patients for GBC Plasma Dysregulated Proteins. [file 12014_2023_9399_MOESM8_ESM.docx]

Table S1: 96-Deep Well Plate Setup

| Row A | 50 mM ammonium bicarbonate (Ambic) pH 8 with Trypsin – 200 µl total |
| --- | --- |
|  |  |
| Row B | 70% Ethanol – 500 µl |
| Row C | 70% Ethanol – 500 µl |
| Row D | 95% Acetonitrile – 500 µl |
| Row E | 95% Acetonitrile – 500 µl |
| Row F | 95% Acetonitrile – 500 µl |
| Row G | Beads (minimum 5 µl)/Protein (up to 25 µl)/Acetonitrile (30% volume of beads + protein/70% of 100% Acetonitrile) – minimum 100 µl |
| Row H | Disposable plastic comb |

Table S2: Concatenation of Fractions from High pH RP Fractionation

| Concatenation | Fractions |
| --- | --- |
| 1 | F2-4 + F12 |
| 2 | F5 + F13 |
| 3 | F6 + F14 |
| 4 | F7 + F15 |
| 5 | F8 + F16 |
| 6 | F9 + F17 |
| 7 | F10 + F18 |
| 8 | F11 + F19-21 |

Table S3: Clinical and Demographic Characteristics of Gallbladder Cancer and Gallstone Disease Patients

| **Parameter** | **Normal Ranges*** | **Gallstone Disease (n=13)  Median  [IQR]** | **Gallbladder cancer (n=24**)  Median  [IQR]** | **p-value** |
| --- | --- | --- | --- | --- |
| **Sex   Female, n (%)  Male, n (%)** | - | 11 (84.62%)  2 (15.38%) | 16 (33.33%)  8 (66.67%) | 0.4395^#^ |
| **Age (years)** | - | 43  [36 – 62] | 59.5  [55 – 69] | 0.0078 |
| **White Cell Count (x10^9^/L)** | 3.9 – 12.6 | 7.48  [5.83 – 9.27] | 10.975  [10.10 – 17.06] | <0.001 |
| **Haemoglobin (g/dL)** | 12.4 – 16.7 | 11.9  [10.5 – 12.6] | 10.15  [9.33 – 11.51] | 0.0467 |
| **C-reactive protein (mg/L)** | <5 | 11  [1 – 142] | 129  [61.5 – 192]  (n=23) | 0.0250 |
| **Creatinine (µmol/L)** | 44 – 80 | 65  [59 – 67] | 71.5  [59 – 87.75] | 0.1918 |
| *Liver Function Tests* | | | | |
| **Total bilirubin**  **(µmol/L)** | 2 – 21 | 9  [8 – 21] | 114.5  [18.75 – 191.50] | <0.001 |
| **Direct bilirubin**  **(µmol/L)** | <3.4 | 4  [3 – 14] | 85  [13 – 179] | 0.0011 |
| **Total protein**  **(g/L)** | 60 – 80 | 72  [69 – 75] | 70  [60 – 75.25] | 0.5240 |
| **Albumin**  **(g/L)** | 35 - 52 | 38  [34 – 73] | 31.5  [28.75 – 34.25] | 0.0168 |
| **Alkaline phosphatase (U/L)** | 42 – 98 | 113  [67 – 172] | 615.5  [360.50 – 821.25] | <0.001 |
| **Gamma-glutamyl transferase (U/L)** | 0 – 38 | 76  [22 – 371] | 536  [313 – 740.5] | 0.0044 |
| **Alanine transaminase (U/L)** | 7 – 35 | 49  [24 – 91] | 66  [24.75 – 93.75] | 0.6331 |
| **Aspartate transaminase (U/L)** | 13 – 35 | 39  [26 – 69] | 76.5  [43.25 – 104.50] | 0.0433 |
| **Jaundice** Yes, n (%)  No, n (%) | - | 4 (30.77%)  9 (69.23%) | 20 (83.33%)  4 (16.67%) | 0.0030^#^ |
| **Cholangitis** Yes, n (%)  No, n (%)  Data missing | - | 0 (0%)  6 (46.15%)  7 (53.85%) | 1 (4.17%)  13 (54.17%)  10 (41.67%) | 0.825^#^ |
| **Tumour Type**  Adenocarcinoma  Small neuroendocrine carcinoma  Squamous cell carcinoma  Unknown | - | - | 17 (70.83%)  1 (4.17%)    2 (8.33%)  4 (16.67%) | - |
| **Carbohydrate antigen 19-9 (U/mL)** | 0 – 37 | 1.935  [1.593 – 2.195]  (n=4) | 410  [35.75 – 1204.75]  (n=8) | 0.0081 |

^#^The Fisher’s Exact Test was performed on the sex, jaundice, and cholangitis data. For clinical data and liver function tests, the Mann-Whitney U Test was performed. *Normal physiological ranges obtained from Bio Analytical Research Corporation South Africa (retrieved from https://www.barcsa.co.za/test-directory/test-reference-ranges/, accessed on 21/11/2021, based on female physiological ranges due to majority sample numbers). **Three patients from the gallbladder cancer group were excluded due to missing clinical data information. Hyphen (-) indicates the absence of data.

Table S4: Clinical and Demographic Characteristics of Gallbladder Cancer and Benign Biliary Pathology Plasma Patients

| **Parameter** | **Normal Ranges*** | **Benign Biliary Pathology**  **(n=73)  Median  [IQR]** | **Gallbladder cancer (n=54)  Median  [IQR]** | **p-value** |
| --- | --- | --- | --- | --- |
| **Sex   Female, n (%)  Male, n (%)** | - | 60 (82.19%)  13 (17.81%) | 29 (53.70%)  25 (46.30%) | <0.001^#^ |
| **Age (years)** | - | 39  [31 – 54.5] | 57.5  [47.25 – 66.75] | <0.001 |
| **White Cell Count (x10^9^/L)** | 3.9 – 12.6 | 7.71  [6.53 – 9.09]  (n=60) | 10.47  [7.55 – 13.60]  (n=51) | 0.0182 |
| **Haemoglobin (g/dL)** | 12.4 – 16.7 | 12.25  [10.70 – 13.70]  (n=60) | 10.2  [8.65 – 12.40]  (n=51) | <0.001 |
| **C-reactive protein (mg/L)** | <5 | 15  [8 – 65]  (n=69) | 106  [40 – 193]  (n=54) | <0.001 |
| **Creatinine (µmol/L)** | 44 – 80 | 70  [62 – 82]  (n=61) | 62  [51.5 – 88]  (n=51) | 0.0531 |
| *Liver Function Tests* | | | | |
| **Total bilirubin**  **(µmol/L)** | 2 – 21 | 31  [11 – 97] | 141  [44.50 – 281.75] | <0.001 |
| **Direct bilirubin**  **(µmol/L)** | <3.4 | 23  [4 – 81] | 122  [46 – 239.5] | <0.001 |
| **Total protein**  **(g/L)** | 60 – 80 | 73  [68 – 79]  (n=55) | 63.5  [58 – 72.25]  (n=48) | <0.001 |
| **Albumin**  **(g/L)** | 35 - 52 | 39  [35.5 – 42.0]  (n=55) | 30  [26 – 34.5]  (n=48) | <0.001 |
| **Alkaline phosphatase (U/L)** | 42 – 98 | 192  [112 – 393] | 516.5  [281.25 – 908.75] | <0.001 |
| **Gamma-glutamyl transferase (U/L)** | 0 – 38 | 166  [80 – 419] | 435.5  [238.5 – 860] | <0.001 |
| **Alanine transaminase (U/L)** | 7 – 35 | 45  [17 – 102]  (n=55) | 57.5  [35.75 – 103.25]  (n=48) | 0.2138 |
| **Aspartate transaminase (U/L)** | 13 – 35 | 53  [28 – 90]  (n=55) | 104.5  [44.75 – 185.50]  (n=48) | 0.0060 |
| **Tumour Staging**  I  II  III  IV  Unknown | - | - | 8 (14.29%)  5 (8.93%)  7 (12.50%)  13 (23.21%)  23 (38.89%) | - |
| **Tumour Type**  Adenocarcinoma  Small neuroendocrine carcinoma  Squamous cell carcinoma  Unknown | - | - | 18 (33.33%)  0 (0%)   1 (1.85%)  35 (64.81%) | - |
| **Carbohydrate antigen 19-9 (U/mL)** | 0 – 37 | 113  [70.5 – 184.5]  (n=3) | 364  [25 – 1479]  (n=18) | 0.6509 |

^#^The Fisher’s Exact Test was performed on the sex data. For clinical data and liver function tests, the Mann-Whitney U Test was performed. *Normal physiological ranges obtained from Bio Analytical Research Corporation South Africa (retrieved from https://www.barcsa.co.za/test-directory/test-reference-ranges/, accessed on 21/11/2021, based on female physiological ranges due to majority sample numbers). Hyphen (-) indicates the absence of data.

Table S5: TNM Staging and Gallstone Disease History for GBC Plasma Patients

| Patient Number | TNM Staging | Gallstone History |
| --- | --- | --- |
| 1 | T4 N2 M1 | No |
| 2 | T4 N2 M1 | Yes |
| 3 | Unknown | Unknown |
| 4 | T4 N2 M1 | Unknown |
| 5 | Unknown | Yes |
| 6 | Unknown | Yes |
| 7 | T2a N1 M1 | Yes |
| 8 | Unknown | No |
| 9 | T1 N0 M0 | No |
| 10 | T3 N0 M0 | Unknown |
| 11 | T3 N0 M0 | Unknown |
| 12 | T3 N1 M1 | No |
| 13 | T2b N1 M0 | Unknown |
| 14 | T2b N1 M1 | Yes |
| 15 | T3 N0 M0 | Unknown |
| 16 | T3 N0 M0 | No |
| 17 | T2b N1 M0 | Unknown |
| 18 | T1 | No |
| 19 | Unknown | Yes |
| 20 | T2b | Unknown |
| 21 | Unknown | No |
| 22 | Unknown | No |
| 23 | Unknown | Yes |
| 24 | Unknown | Unknown |
| 25 | T3 N1 M1 | No |
| 26 | Unknown | Unknown |
| 27 | Unknown | Yes |
| 28 | Unknown | Yes |
| 29 | Unknown | No |
| 30 | Unknown | No |
| 31 | T4 | No |
| 32 | T3 N1 Mx | No |
| 33 | T4 | No |
| 34 | Unknown | Yes |
| 35 | T4 | No |
| 36 | T4 | No |
| 37 | Unknown | Yes |
| 38 | T4 N2 M1 | No |
| 39 | T4 | No |
| 40 | T1 | No |
| 42 | T1 | No |
| 43 | T1 | No |
| 44 | T4 N2 Mx | Yes |
| 45 | T1 | No |
| 46 | Unknown | No |
| 47 | T1 | No |
| 48 | T4 | No |
| 49 | T4 | Yes |
| 50 | T4 N2 M0 | No |
| 51 | T1 | Yes |
| 52 | Unknown | Unknown |
| 53 | Unknown | Unknown |
| 55 | Unknown | Unknown |
| 56 | Unknown | Unknown |

Table S6: The Total Precursors, Peptides, and Protein Groups Identified for Each Individual Patient for The GBC Versus Normal Comparison

| Sample number | Precursors | Peptides | Protein Groups |
| --- | --- | --- | --- |
| Normal 1 | 13,787 | 11,276 | 1,813 |
| Normal 2 | 14,787 | 12,108 | 1,869 |
| Normal 3 | 18,102 | 14,760 | 2,116 |
| Normal 4 | 12,269 | 10,042 | 1,680 |
| Normal 5 | 12,968 | 10,543 | 1,749 |
| GBC 7 | 10,697 | 8,692 | 1,605 |
| GBC 8 | 22,169 | 18,111 | 2,349 |
| GBC 11 | 12,654 | 10,528 | 1,801 |
| GBC 12 | 8,768 | 7,202 | 1,320 |
| GBC 13 | 16,236 | 12,998 | 1,957 |
| GBC 14 | 24,683 | 19,538 | 2,413 |
| GBC 15 | 22,469 | 17,795 | 2,415 |
| GBC 16 | 24,670 | 19,762 | 2,457 |
| GBC 17 | 18,541 | 14,956 | 2,161 |
| GBC 18 | 11,367 | 9,482 | 1,674 |
| GBC 19 | 19,298 | 15,580 | 2,159 |
| GBC 21 | 22,882 | 17,873 | 2,258 |
| GBC 22 | 23,240 | 18,334 | 2,338 |
| GBC 24 | 20,129 | 16,169 | 2,145 |
| GBC 25 | 21,035 | 16,673 | 2,259 |
| GBC 26 | 21,206 | 16,754 | 2,192 |
| GBC 27 | 12,779 | 10,327 | 1,701 |
| GBC 28 | 21,113 | 16,615 | 2,284 |
| GBC 29 | 21,030 | 16,689 | 2,245 |
| GBC 30 | 10,382 | 8,452 | 1,583 |
| GBC 31 | 18,048 | 14,368 | 2,001 |
| GBC 32 | 19,162 | 15,315 | 2,214 |
| GBC 33 | 13,757 | 11,461 | 1,943 |
| GBC 34 | 11,388 | 9,301 | 1,688 |
| GBC 35 | 13,199 | 10,534 | 1,629 |
| GBC 36 | 24,206 | 19,161 | 2,430 |
| GBC 37 | 21,250 | 17,136 | 2,339 |
| GBC = Gallbladder Cancer | | | |

Table S7: The Total Precursors, Peptides, and Protein Groups Identified for Each Individual Patient for The GBC Versus GD Comparison

| Sample number | Precursors | Peptides | Protein Groups |
| --- | --- | --- | --- |
| GD 1 | 12,737 | 10,125 | 1,623 |
| GD 2 | 6,375 | 5,128 | 958 |
| GD 3 | 11,297 | 9,099 | 1,472 |
| GD 4 | 8,701 | 7,031 | 1,203 |
| GD 5 | 10,836 | 8,758 | 1,538 |
| GD 6 | 14,447 | 11,514 | 1,802 |
| GD 7 | 13,195 | 10,688 | 1,758 |
| GD 9 | 18,144 | 14,182 | 1,993 |
| GD 10 | 11,417 | 9,157 | 1,446 |
| GD 11 | 13,248 | 10,413 | 1,581 |
| GD 12 | 16,728 | 13,401 | 2,013 |
| GD 14 | 15,737 | 12,323 | 1,793 |
| GD 15 | 12,518 | 9,927 | 1,612 |
| GBC 7 | 10,699 | 8,693 | 1,545 |
| GBC 8 | 22,129 | 18,073 | 2,338 |
| GBC 11 | 12,663 | 10,536 | 1,809 |
| GBC 12 | 8,768 | 7,201 | 1,319 |
| GBC 13 | 16,239 | 13,000 | 1,957 |
| GBC 14 | 24,647 | 19,502 | 2,400 |
| GBC 15 | 22,470 | 17,795 | 2,414 |
| GBC 16 | 24,653 | 19,744 | 2,450 |
| GBC 17 | 18,551 | 14,966 | 2,163 |
| GBC 18 | 11,360 | 9,474 | 1,671 |
| GBC 19 | 19,301 | 15,581 | 2,159 |
| GBC 21 | 22,882 | 17,872 | 2,245 |
| GBC 22 | 23,220 | 18,313 | 2,332 |
| GBC 24 | 20,115 | 16,154 | 2,142 |
| GBC 25 | 21,040 | 16,677 | 2,263 |
| GBC 26 | 21,099 | 16,745 | 2,191 |
| GBC 27 | 12,785 | 10,333 | 1,707 |
| GBC 28 | 21,105 | 16,605 | 2,281 |
| GBC 29 | 21,035 | 16,693 | 2,244 |
| GBC 30 | 10,390 | 8,459 | 1,589 |
| GBC 31 | 18,053 | 14,372 | 2,006 |
| GBC 32 | 19,163 | 15,314 | 2,210 |
| GBC 33 | 13,755 | 11,459 | 1,945 |
| GBC 34 | 11,387 | 9,300 | 1,687 |
| GBC 35 | 13,203 | 10,537 | 1,631 |
| GBC 36 | 21,187 | 19,141 | 2,423 |
| GBC 37 | 21,223 | 17,107 | 2,326 |
| GD = Gallstone Disease  GBC = Gallbladder Cancer | | | |

Table S8: The Total Precursors, Peptides, and Protein Groups Identified for Each Individual Patient for The GBC Versus BBP Plasma Comparison

| Sample number | Precursors | Peptides | Protein Groups |
| --- | --- | --- | --- |
| BBP 1 | 3,915 | 2,618 | 232 |
| BBP 2 | 3,714 | 2,501 | 225 |
| BBP 3 | 3,516 | 2,381 | 220 |
| BBP 4 | 3,992 | 2,654 | 237 |
| BBP 5 | 3,673 | 2,461 | 228 |
| BBP 6 | 3,854 | 2,544 | 230 |
| BBP 9 | 3,666 | 2,479 | 224 |
| BBP 10 | 3,754 | 2,504 | 229 |
| BBP 11 | 3,809 | 2,552 | 226 |
| BBP 12 | 3,981 | 2,612 | 230 |
| BBP 13 | 3,826 | 2,554 | 235 |
| BBP 14 | 4,082 | 2,704 | 244 |
| BBP 15 | 3,695 | 2,488 | 230 |
| BBP 16 | 3,639 | 2,442 | 226 |
| BBP 17 | 3,836 | 2,567 | 232 |
| BBP 18 | 3,807 | 2,519 | 229 |
| BBP 19 | 4,099 | 2,686 | 229 |
| BBP 23 | 3,789 | 2,536 | 236 |
| BBP 24 | 3,746 | 2,509 | 231 |
| BBP 25 | 3,890 | 2,594 | 237 |
| BBP 26 | 3,717 | 2,497 | 221 |
| BBP 27 | 3,816 | 2,544 | 232 |
| BBP 28 | 3,863 | 2,587 | 234 |
| BBP 30 | 3,629 | 2,451 | 240 |
| BBP 31 | 3,807 | 2,515 | 234 |
| BBP 32 | 3,613 | 2,438 | 222 |
| BBP 33 | 3,925 | 2,575 | 227 |
| BBP 34 | 3,969 | 2,615 | 234 |
| BBP 35 | 3,747 | 2,484 | 225 |
| BBP 36 | 3,958 | 2,594 | 232 |
| BBP 37 | 3,737 | 2,509 | 238 |
| BBP 38 | 3,569 | 2,402 | 225 |
| BBP 39 | 3,845 | 2,552 | 234 |
| BBP 40 | 4,122 | 2,681 | 229 |
| BBP 41 | 3,957 | 2,582 | 232 |
| BBP 42 | 4,088 | 2,671 | 246 |
| BBP 43 | 4,070 | 2,638 | 227 |
| BBP 44 | 4,062 | 2,661 | 239 |
| BBP 45 | 4,012 | 2,642 | 245 |
| BBP 46 | 4,230 | 2,753 | 238 |
| BBP 47 | 3,999 | 2,626 | 236 |
| BBP 48 | 4,034 | 2,649 | 238 |
| BBP 49 | 4,043 | 2,648 | 230 |
| BBP 50 | 3,910 | 2,574 | 233 |
| BBP 51 | 3,950 | 2,610 | 239 |
| BBP 52 | 3,790 | 2,503 | 227 |
| BBP 53 | 3,859 | 2,541 | 236 |
| BBP 54 | 3,696 | 2,460 | 232 |
| BBP 55 | 3,864 | 2,546 | 231 |
| BBP 56 | 3,853 | 2,534 | 230 |
| BBP 57 | 4,123 | 2,681 | 235 |
| BBP 58 | 3,879 | 2,567 | 229 |
| BBP 59 | 4,002 | 2,633 | 233 |
| BBP 60 | 3,874 | 2,590 | 237 |
| BBP 61 | 3,883 | 2,560 | 227 |
| BBP 62 | 3,829 | 2,530 | 227 |
| BBP 63 | 4,062 | 2,647 | 235 |
| BBP 64 | 4,049 | 2,617 | 229 |
| BBP 65 | 3,739 | 2,463 | 224 |
| BBP 67 | 3,664 | 2,429 | 216 |
| BBP 68 | 3,809 | 2,513 | 227 |
| BBP 70 | 3,766 | 2,479 | 224 |
| BBP 71 | 3,551 | 2,372 | 225 |
| BBP 72 | 3,539 | 2,323 | 224 |
| BBP 73 | 3,785 | 2,485 | 222 |
| BBP 74 | 3,395 | 2,250 | 215 |
| BBP 75 | 3,704 | 2,454 | 228 |
| BBP 76 | 3,746 | 2,460 | 231 |
| BBP 77 | 3,499 | 2,313 | 221 |
| BBP 78 | 3,182 | 2,167 | 216 |
| BBP 79 | 3,897 | 2,562 | 228 |
| BBP 80 | 4,030 | 2,641 | 240 |
| BBP 83 | 3,727 | 2,454 | 216 |
| GBC 1 | 3,850 | 2,569 | 227 |
| GBC 2 | 4,016 | 2,656 | 229 |
| GBC 3 | 3,963 | 2,639 | 240 |
| GBC 4 | 3,713 | 2,500 | 229 |
| GBC 5 | 3,932 | 2,592 | 231 |
| GBC 6 | 3,740 | 2,488 | 224 |
| GBC 7 | 3,895 | 2,590 | 224 |
| GBC 8 | 3,741 | 2,530 | 234 |
| GBC 9 | 3,854 | 2,561 | 226 |
| GBC 10 | 3,970 | 2,635 | 229 |
| GBC 11 | 3,991 | 2,638 | 232 |
| GBC 12 | 3,964 | 2,618 | 227 |
| GBC 13 | 3,833 | 2,566 | 227 |
| GBC 14 | 4,035 | 2,666 | 233 |
| GBC 15 | 3,931 | 2,585 | 215 |
| GBC 16 | 3,826 | 2,545 | 224 |
| GBC 17 | 3,732 | 2,520 | 230 |
| GBC 18 | 3,813 | 2,569 | 238 |
| GBC 19 | 3,722 | 2,467 | 225 |
| GBC 20 | 3,783 | 2,538 | 238 |
| GBC 21 | 3,893 | 2,585 | 221 |
| GBC 22 | 3,783 | 2,514 | 224 |
| GBC 23 | 3,994 | 2,616 | 221 |
| GBC 24 | 3,884 | 2,562 | 236 |
| GBC 25 | 3,934 | 2,593 | 230 |
| GBC 26 | 3,627 | 2,419 | 227 |
| GBC 27 | 3,976 | 2,605 | 228 |
| GBC 28 | 4,155 | 2,679 | 238 |
| GBC 29 | 3,675 | 2,441 | 220 |
| GBC 30 | 4,026 | 2,659 | 231 |
| GBC 31 | 4,192 | 2,715 | 242 |
| GBC 32 | 4,060 | 2,642 | 231 |
| GBC 33 | 3,890 | 2,555 | 228 |
| GBC 34 | 3,958 | 2,623 | 246 |
| GBC 35 | 4,141 | 2,716 | 234 |
| GBC 36 | 4,161 | 2,693 | 242 |
| GBC 37 | 4,101 | 2,676 | 229 |
| GBC 38 | 4,171 | 2,714 | 240 |
| GBC 39 | 4,184 | 2,734 | 240 |
| GBC 40 | 4,094 | 2,700 | 240 |
| GBC 42 | 4,007 | 2,631 | 235 |
| GBC 43 | 4,005 | 2,630 | 233 |
| GBC 44 | 3,996 | 2,615 | 237 |
| GBC 45 | 3,692 | 2,430 | 223 |
| GBC 46 | 3,734 | 2,468 | 234 |
| GBC 47 | 3,760 | 2,453 | 232 |
| GBC 48 | 3,620 | 2,388 | 216 |
| GBC 49 | 3,786 | 2,488 | 220 |
| GBC 50 | 3,728 | 2,451 | 225 |
| GBC 51 | 3,933 | 2,542 | 227 |
| GBC 52 | 3,946 | 2,566 | 227 |
| GBC 53 | 3,593 | 2,403 | 226 |
| GBC 55 | 3,952 | 2,577 | 230 |
| GBC 56 | 3,914 | 2,588 | 236 |
| BBP = Benign Biliary Pathology  GBC = Gallbladder Cancer | | | |

Table S9: All Identified Dysregulated Proteins in GBC Compared to Normal

| Protein Group | Protein Name | No. of Unique Peptides | Average Log_2_ Ratio | p-value | q-value |
| --- | --- | --- | --- | --- | --- |
| Q15063 | POSTN – Periostin | 59 | 5.02066359 | <0.001 | <0.001 |
| P04732 | MT1E – Metallothionein 1E | 3 | 4.712206662 | <0.001 | <0.001 |
| P31327 | CPSM – Carbamoyl-phosphate synthase | 103 | 4.483369762 | <0.001 | <0.001 |
| P07148 | FABPL – Fatty acid binding protein | 14 | 4.024563036 | <0.001 | <0.001 |
| Q99715 | COCA1 – Collagen alpha-1(XII) chain | 143 | 4.004558467 | <0.001 | <0.001 |
| P02792 | FRIL – Ferritin light chain | 11 | 3.964146323 | <0.001 | <0.001 |
| P04004 | VTNC – Vitronectin | 18 | 3.915829431 | <0.001 | <0.001 |
| P54868 | HMCS2 – Hydroxymethylglutaryl-CoA synthase | 26 | 3.915381366 | <0.001 | <0.001 |
| P52895 | AK1C2 – Aldo-keto reductase family 1 member C2 | 5 | 3.848081936 | <0.001 | <0.001 |
| P52758 | RIDA – 2-iminobutanoate/2-iminopropanoate deaminase | 6 | 3.70928337 | <0.001 | <0.001 |
| P04908 | H2A1B – Histone H2A type 1-B/E | 2 | 3.601816369 | <0.001 | <0.001 |
| Q7L7L0 | H2A3 – Histone H2A type 3 | 2 | 3.601816369 | <0.001 | <0.001 |
| Q93077 | H2A1C – Histone H2A type 1-C | 2 | 3.601816369 | <0.001 | <0.001 |
| P07996 | TSP – Thrombospondin-1 | 54 | 3.251710872 | <0.001 | <0.001 |
| P04264 | KSC1 – Keratin 1 | 54 | 3.25004815 | <0.001 | <0.001 |
| P12004 | PCNA – Proliferating cell nuclear antigen | 10 | 3.172973883 | <0.001 | <0.001 |
| P02751 | FINC – Fibronectin | 117 | 3.0986923 | <0.001 | <0.001 |
| Q7Z4W1 | DCXR – L-xylulose reductase | 13 | 2.983416149 | <0.001 | <0.001 |
| P02649 | APOE – Apolipoprotein E | 26 | 2.968525542 | <0.001 | <0.001 |
| Q06278 | AOXA – Aldehyde oxidase 1 | 43 | 2.942234669 | <0.001 | <0.001 |
| P07477 | TRY1 – Serine protease 1 | 2 | 2.931102462 | <0.001 | <0.001 |
| Q02338 | BDH – 3-hydroxybutyrate dehydrogenase 1 | 15 | 2.900769273 | <0.001 | <0.001 |
| P01833 | PIGR – Polymeric immunoglobulin receptor | 29 | 2.898754081 | <0.001 | <0.001 |
| P01911 | DRB1 – HLA class II histocompatibility antigen, DRB1 beta chain | 10 | 2.740000646 | <0.001 | <0.001 |
| P15153 | RAC2 – Rac family small GTPase 2 | 5 | 2.737986073 | <0.001 | <0.001 |
| P05413 | FABPH – Fatty acid binding protein | 11 | 2.731079529 | <0.01 | <0.01 |
| P24821 | TENA – Tenascin C | 84 | 2.712643782 | <0.001 | <0.001 |
| P13611 | CSPG2 – Versican core protein | 31 | 2.635654171 | <0.001 | <0.001 |
| P35555 | FBN1 – Fibrillin-1 | 108 | 2.619872137 | <0.001 | <0.001 |
| Q08426 | ECHP – Peroxisomal bifunctional enzyme | 39 | 2.608263782 | <0.001 | <0.001 |
| P00747 | PLMN – Plasminogen | 54 | 2.587929691 | <0.001 | <0.001 |
| P19971 | TYPH – Thymidine phosphorylase | 25 | 2.561671217 | <0.001 | <0.001 |
| P34897 | GLYM – Serine hydroxymethyltransferase 2 | 21 | 2.51078038 | <0.001 | <0.001 |
| P02452 | CO1A1 – Collagen alpha-1(I) chain | 29 | 2.507681268 | <0.001 | <0.001 |
| P02748 | CO9 – Complement C9 | 26 | 2.454653675 | <0.001 | <0.001 |
| P11215 | ITAM – Integrin subunit alpha M | 31 | 2.441464589 | <0.001 | <0.001 |
| Q00688 | FKBP3 – Integrin alpha-M | 11 | 2.427845643 | <0.001 | <0.001 |
| P09110 | THIK - Acetyl-CoA acyltransferase | 17 | 2.383977059 | <0.001 | <0.001 |
| P21333 | FLNA – Filamin-A | 169 | -2.489748582 | <0.001 | <0.001 |
| P62736 | ACTA – Actin alpha 2 | 21 | -2.492619937 | <0.001 | <0.001 |
| P09493 | TPM1 – Tropomyosin alpha-1 chain | 25 | -2.60007637 | <0.01 | <0.01 |
| Q03135 | CAV1 – Caveolin 1 | 9 | -2.651299406 | <0.001 | <0.001 |
| P51911 | CNN1 – Calponin 1 | 27 | -2.741320166 | <0.001 | <0.001 |
| P18085 | ARF4 – ADP ribosylation factor 4 | 6 | -2.756697069 | <0.001 | <0.001 |
| P09525 | ANXA4 – Annexin A4 | 36 | -2.787707074 | <0.01 | <0.01 |
| P10301 | RRAS – Ras-related protein R-Ras | 14 | -2.788038724 | <0.001 | <0.001 |
| P20231 | TRYB2 – Tryptase beta 2 | 16 | -2.800721074 | <0.001 | <0.001 |
| Q15661 | TRYB1 – Tryptase alpha/beta 1 | 16 | -2.800721074 | <0.001 | <0.001 |
| P20774 | MIME – Mimecan | 23 | -2.895872701 | <0.001 | <0.001 |
| P07951 | TPM2 – Tropomyosin beta chain | 26 | -2.980807556 | <0.001 | <0.001 |
| Q9HBI1 | PARVB – Beta-parvin | 3 | -3.106309788 | <0.001 | <0.001 |
| P00918 | CAH2 – Carbonic anhydrase 2 | 18 | -3.359399413 | <0.001 | <0.001 |
| P06703 | S10A6 – Protein S100-A6 | 9 | -3.362875006 | <0.001 | <0.001 |
| Q01995 | TAGL – Transgelin | 31 | -3.372011937 | <0.01 | <0.01 |
| P17661 | DESM – Desmin | 53 | -3.441316866 | <0.001 | <0.001 |
| Q9NZN4 | EHD2 – EH domain-containing protein 2 | 36 | -3.475631992 | <0.001 | <0.001 |
| O14950 | ML12B – Myosin regulatory light chain 12B | 10 | -3.549636751 | <0.001 | <0.001 |
| P19105 | ML12A – Myosin regulatory light chain 12A | 10 | -3.549636751 | <0.001 | <0.001 |
| P24844 | MYL9 – Myosin regulatory light polypeptide 9 | 6 | -3.628772097 | <0.001 | <0.001 |
| P35749 | MYH11 – Myosin-11 | 231 | -3.652336209 | <0.001 | <0.001 |
| Q6NZI2 | CAVN1 – Caveolae-associated protein 1 | 19 | -3.822693564 | <0.001 | <0.001 |
| O60271 | JIP4 – C-Jun-amino-terminal kinase-interacting protein 4 | 24 | -4.856406351 | <0.01 | <0.01 |

Table S10: All Identified Dysregulated Proteins in GBC Compared to GD

| Protein Group | Protein Name | No. of Unique Peptides | | | Average Log_2_ Ratio | p-value | q-value |
| --- | --- | --- | --- | --- | --- | --- | --- |
| P52895 | AK1C2 – Aldo-keto reductase family 1 member C2 | | 5 | 4.953323214 | | <0.001 | <0.001 |
| P31327 | CPSM – Carbamoyl-phosphate synthase | | 103 | 4.323381454 | | <0.001 | <0.001 |
| P11498 | PYC – Pyruvate carboxylase | | 56 | 4.065746017 | | <0.001 | <0.001 |
| Q04828 | AK1C1 – Aldo-keto reductase family 1 member C1 | | 23 | 3.67721375 | | <0.001 | <0.001 |
| Q02338 | BDH – D-beta-hydroxybutyrate dehydrogenase | | 15 | 3.638123672 | | <0.001 | <0.001 |
| P52758 | RIDA – 2-iminobutanoate/2-iminopropanoate deaminase | | 6 | 3.587142384 | | <0.001 | <0.001 |
| Q99715 | COCA1 – Collagen alpha-1(XII) chain | | 143 | 3.374782172 | | <0.001 | <0.001 |
| O60701 | UGDH – UDP-glucose 6-dehydrogenase | | 31 | 3.088888829 | | <0.001 | <0.001 |
| P07996 | TSP1 – Thrombospondin-1 | | 54 | 3.003882122 | | <0.001 | <0.001 |
| Q7Z4W1 | DCXR – L-xylulose reductase | | 13 | 2.844273258 | | <0.001 | <0.001 |
| P35442 | TSP2 – Thrombospondin-2 | | 37 | 2.780811447 | | <0.001 | <0.001 |
| P42765 | THIM – 3-ketoacyl-CoA thiolase | | 31 | 2.691117194 | | <0.001 | <0.001 |
| P05062 | ALDOB – Fructose-bisphosphate aldolase B | | 26 | 2.64136246 | | <0.001 | <0.001 |
| P42330 | AK1C3 – Aldo-keto reductase family 1 member C3 | | 12 | 2.626561093 | | <0.001 | <0.001 |
| Q8IUX7 | AEBP1 – Adipocyte enhancer-binding protein 1 | | 24 | 2.608124891 | | <0.001 | <0.001 |
| Q14956 | GPNMB – Transmembrane glycoprotein NMB | | 2 | 2.592714206 | | <0.001 | <0.001 |
| P16219 | ACADS – Short-chain specific acyl-CoA dehydrogenase | | 17 | 2.557209494 | | <0.001 | <0.001 |
| P12004 | PCNA – Proliferating cell nuclear antigen | | 10 | 2.479580402 | | <0.001 | <0.001 |
| P06731 | CEAM5 – Carcinoembryonic antigen-related cell adhesion molecule 5 | | 5 | 2.468455016 | | <0.001 | <0.001 |
| Q96HE7 | ERO1A – ERO1-like protein alpha | | 23 | 2.418763344 | | <0.001 | <0.001 |
| Q08380 | LG3BP – Galectin-3-binding protein | | 17 | 2.371437513 | | <0.001 | <0.001 |
| O60218 | AK1BA – Aldo-keto reductase family 1 member B10 | | 26 | 2.356581673 | | <0.001 | <0.001 |
| Q15063 | POSTN – Periostin | | 59 | 2.216168479 | | <0.001 | <0.001 |
| P01833 | PIGR – Polymeric immunoglobulin receptor | | 29 | 2.196173212 | | <0.001 | <0.001 |
| P00966 | ASSY – Argininosuccinate synthase | | 21 | 2.176407723 | | <0.001 | <0.001 |
| P11413 | G6PD – Glucose-6-phosphate 1-dehydrogenase | | 40 | 2.17522764 | | <0.001 | <0.001 |
| P08263 | GSTA1 – Glutathione S-transferase A1 | | 31 | 2.172464657 | | <0.001 | <0.001 |
| P16930 | FAAA – Fumarylacetoacetase | | 15 | 2.127056816 | | <0.001 | <0.001 |
| P23141 | EST1 – Liver carboxylesterase 1 | | 36 | 2.109240352 | | <0.001 | <0.001 |
| P02649 | APOE – Apolipoprotein E | | 26 | 2.088429222 | | <0.001 | <0.001 |
| P51659 | DHB4 – Peroxisomal multifunctional enzyme type 2 | | 36 | 2.081996564 | | <0.001 | <0.001 |
| P00167 | CYB5 – Cytochrome b5 | | 8 | 2.076020282 | | <0.001 | <0.001 |
| Q30154 | DRB5 – HLA class II histocompatibility antigen | | 3 | 2.07583031 | | <0.01 | <0.01 |
| P09467 | F16P1 – Fructose-1,6-bisphosphatase 1 | | 17 | 2.063445957 | | <0.001 | <0.001 |
| P08729 | K2C7 – Keratin, type II cytoskeletal 7 | | 48 | 2.054256483 | | <0.001 | <0.001 |
| P17174 | AATC – Aspartate aminotransferase | | 26 | 2.046466287 | | <0.001 | <0.001 |
| Q16698 | DECR – 2,4-dienoyl-CoA reductase | | 18 | 2.016535491 | | <0.001 | <0.001 |
| Q16881 | TRXR1 – Thioredoxin reductase 1 | | 19 | 1.980805794 | | <0.001 | <0.001 |
| P05783 | K1C18 – Keratin, type I cytoskeletal 18 | | 37 | 1.975434726 | | <0.001 | <0.001 |
| P10809 | CH60 – 60 kDa heat shock protein | | 54 | 1.920904009 | | <0.001 | <0.001 |
| P24752 | THIL – Acetyl-CoA acetyltransferase | | 33 | 1.914611807 | | <0.001 | <0.001 |
| P16949 | STMN1 – Stathmin | | 6 | 1.895356424 | | <0.001 | <0.001 |
| P34897 | GLYM – Serine hydroxymethyltransferase | | 21 | 1.862362934 | | <0.001 | <0.001 |
| P16435 | NCPR – NADPH--cytochrome P450 reductase | | 27 | 1.848717579 | | <0.001 | <0.001 |
| P59665 | DEF1 – Neutrophil defensin 1 | | 4 | 1.838381823 | | <0.001 | <0.001 |
| P59666 | DEF3 – Neutrophil defensin 3 | | 4 | 1.838381823 | | <0.001 | <0.001 |
| P00367 | DHE3 – Glutamate dehydrogenase 1 | | 33 | 1.830040693 | | <0.001 | <0.001 |
| O95831 | AIFM1 – Apoptosis-inducing factor 1 | | 27 | 1.805722015 | | <0.001 | <0.001 |
| P19971 | TYPH – Thymidine phosphorylase | | 25 | 1.795493292 | | <0.001 | <0.001 |
| P38117 | ETFB – Electron transfer flavoprotein subunit beta | | 21 | 1.791739209 | | <0.001 | <0.001 |
| P30084 | ECHM – Enoyl-CoA hydratase | | 22 | 1.764578278 | | <0.001 | <0.001 |
| P24821 | TENA – Tenascin | | 84 | 1.763552536 | | <0.001 | <0.001 |
| P05787 | K2C8 – Keratin, type II cytoskeletal 8 | | 47 | 1.751221966 | | <0.001 | <0.001 |
| P07858 | CATB – Cathepsin B | | 14 | 1.745793698 | | <0.001 | <0.001 |
| P38646 | GRP75 – Stress-70 protein | | 43 | 1.744767062 | | <0.001 | <0.001 |
| P11586 | C1TC – C-1-tetrahydrofolate synthase | | 55 | 1.742940969 | | <0.001 | <0.001 |
| Q01813 | PFKAP – ATP-dependent 6-phosphofructokinase | | 31 | 1.742191304 | | <0.001 | <0.001 |
| Q01469 | FABP5 – Fatty acid-binding protein 5 | | 21 | 1.736882963 | | <0.05 | <0.01 |
| P08727 | K1C19 – Keratin, type I cytoskeletal 19 | | 30 | 1.703228728 | | <0.001 | <0.001 |
| P05141 | ADT2 – ADP/ATP translocase 2 | | 10 | 1.692862602 | | <0.001 | <0.001 |
| Q9Y2Q3 | GSTK1 – Glutathione S-transferase kappa 1 | | 12 | 1.680971617 | | <0.001 | <0.001 |
| P15153 | RAC2 – Ras-related C3 botulinum toxin substrate 2 | | 5 | 1.675119445 | | <0.001 | <0.001 |
| P08195 | 4F2 – 4F2 cell-surface antigen heavy chain | | 15 | 1.665178905 | | <0.001 | <0.001 |
| P31949 | S10AB – Protein S100-A11 | | 6 | 1.662021978 | | <0.001 | <0.001 |
| P07339 | CATD – Cathepsin D | | 28 | 1.649144014 | | <0.001 | <0.001 |
| Q9BPW8 | NIPS1 – NipSnap homolog 1 | | 9 | 1.626564893 | | <0.01 | <0.01 |
| P21796 | VDAC1 – Voltage-dependent anion-selective channel protein 1 | | 21 | 1.614328869 | | <0.001 | <0.001 |
| P43304 | GPDM – Glycerol-3-phosphate dehydrogenase | | 24 | 1.597357909 | | <0.001 | <0.001 |
| O75874 | IDHC – Isocitrate dehydrogenase | | 26 | 1.595013547 | | <0.001 | <0.001 |
| P07099 | HYEP – Epoxide hydrolase 1 | | 33 | 1.586795424 | | <0.001 | <0.001 |
| P08238 | HS90B – Heat shock protein HSP 90-beta | | 53 | 1.576348982 | | <0.001 | <0.001 |
| P13804 | ETFA – Electron transfer flavoprotein subunit alpha | | 17 | 1.570324464 | | <0.001 | <0.001 |
| Q9H0W9 | CK054 – Ester hydrolase C11orf54 | | 11 | 1.569835702 | | <0.01 | <0.01 |
| P22307 | SCP2 – Sterol carrier protein 2 | | 36 | 1.565157933 | | <0.001 | <0.001 |
| P05107 | ITB2 – Integrin beta-2 | | 32 | 1.563240053 | | <0.001 | <0.001 |
| P48735 | IDHP – Isocitrate dehydrogenase | | 30 | 1.558495224 | | <0.001 | <0.001 |
| O15533 | TPSN – Tapasin | | 6 | 1.558452978 | | <0.001 | <0.001 |
| P01903 | DRA – HLA class II histocompatibility antigen, DR alpha chain | | 8 | 1.552950249 | | <0.01 | <0.01 |
| P40121 | CAPG – Macrophage-capping protein | | 17 | 1.551524099 | | <0.001 | <0.001 |
| P50225 | ST1A1 – Sulfotransferase 1A1 | | 11 | 1.546602828 | | <0.01 | <0.01 |
| P20292 | AL5AP – Arachidonate 5-lipoxygenase-activating protein | | 2 | 1.54098875 | | <0.001 | <0.001 |
| P61604 | CH10 – 10 kDa heat shock protein | | 14 | 1.533125541 | | <0.001 | <0.001 |
| P23229 | ITA6 – Integrin alpha-6 | | 23 | 1.528409047 | | <0.001 | <0.001 |
| P04004 | VTNC – Vitronectin | | 18 | 1.508175969 | | <0.01 | <0.01 |
| Q6YN16 | HSDL2 – Hydroxysteroid dehydrogenase-like protein 2 | | 19 | 1.477875786 | | <0.001 | <0.001 |
| Q13283 | G3BP1 – Ras GTPase-activating protein-binding protein 1 | | 15 | 1.476789382 | | <0.001 | <0.001 |
| Q15185 | TEBP – Prostaglandin E synthase 3 | | 6 | 1.476785049 | | <0.001 | <0.001 |
| P28161 | GSTM2 – Glutathione S-transferase Mu 2 | | 6 | -1.479098041 | | <0.001 | <0.001 |
| O43294 | TGFI1 – Transforming growth factor beta-1-induced transcript 1 protein | | 13 | -1.481158888 | | <0.001 | <0.001 |
| P05090 | APOD – Apolipoprotein D | | 12 | -1.497688594 | | <0.01 | <0.01 |
| P01619 | KV320 – Immunoglobulin kappa variable 3-20 | | 4 | -1.508669451 | | <0.001 | <0.001 |
| P14543 | NID1 – Nidogen-1 | | 34 | -1.512686623 | | <0.001 | <0.001 |
| P04433 | KV311 – Immunoglobulin kappa variable 3-11 | | 2 | -1.531428044 | | <0.01 | <0.01 |
| Q7Z5L7 | PODN – Podocan | | 20 | -1.535722608 | | <0.001 | <0.001 |
| P12109 | CO6A1 – Collagen alpha-1(VI) chain | | 51 | -1.53845217 | | <0.001 | <0.001 |
| P08185 | CBG – Corticosteroid-binding globulin | | 5 | -1.541950634 | | <0.001 | <0.001 |
| P04196 | HRG – Histidine-rich glycoprotein | | 18 | -1.546626855 | | <0.01 | <0.01 |
| Q96AC1 | FERM2 – Fermitin family homolog 2 | | 32 | -1.553301196 | | <0.001 | <0.001 |
| Q9HBI1 | PARVB – Beta-parvin | | 3 | -1.560212729 | | <0.001 | <0.001 |
| Q15404 | RSU1 – Ras suppressor protein 1 | | 11 | -1.562357193 | | <0.001 | <0.001 |
| Q06828 | FMOD – Fibromodulin | | 11 | -1.578801641 | | <0.001 | <0.001 |
| P02787 | TRFE – Serotransferrin | | 74 | -1.583597892 | | <0.001 | <0.001 |
| P11047 | LAMC1 – Laminin subunit gamma-1 | | 67 | -1.59617644 | | <0.001 | <0.001 |
| P04908 | H2A1B – Histone H2A type 1-B/E | | 2 | -1.597290839 | | <0.001 | <0.001 |
| Q7L7L0 | H2A3 – Histone H2A type 3 | | 2 | -1.597290839 | | <0.001 | <0.001 |
| Q93077 | H2A1C – Histone H2A type 1-C | | 2 | -1.597290839 | | <0.001 | <0.001 |
| Q9BZQ8 | NIBA1 – Niban 1 | | 17 | -1.598688541 | | <0.001 | <0.001 |
| P43121 | MUC18 – Cell surface glycoprotein MUC18 | | 17 | -1.598813306 | | <0.001 | <0.001 |
| P12111 | CO6A3 – Collagen alpha-3(VI) chain | | 184 | -1.61580628 | | <0.001 | <0.001 |
| P02790 | HEMO – Hemopexin | | 31 | -1.618488319 | | <0.001 | <0.001 |
| Q13418 | ILK – Integrin-linked protein kinase | | 23 | -1.636966035 | | <0.001 | <0.001 |
| P43652 | AFAM – Afamin | | 23 | -1.650870889 | | <0.001 | <0.001 |
| Q9UBX5 | FBLN5 – Fibulin-5 | | 15 | -1.674528913 | | <0.001 | <0.001 |
| P55083 | MFAP4 – Microfibril-associated glycoprotein 4 | | 6 | -1.674834435 | | <0.001 | <0.001 |
| P01614 | KVD40 – Immunoglobulin kappa variable 2D-40 | | 4 | -1.687001286 | | <0.001 | <0.001 |
| P02753 | RET4 – Retinol-binding protein 4 | | 11 | -1.68830487 | | <0.001 | <0.001 |
| P01871 | IGHM – Immunoglobulin heavy constant mu | | 7 | -1.709143093 | | <0.05 | <0.01 |
| P00568 | KAD1 – Adenylate kinase isoenzyme 1 | | 12 | -1.735247741 | | <0.01 | <0.01 |
| Q13228 | SBP1 – Methanethiol oxidase | | 30 | -1.73889701 | | <0.01 | <0.01 |
| A0A075B6H7 | KV37 – Probable non-functional immunoglobulin kappa variable 3-7 | | 2 | -1.744984995 | | <0.001 | <0.001 |
| P60033 | CD81 – CD81 antigen | | 4 | -1.749183973 | | <0.001 | <0.001 |
| P60660 | MYL6 – Myosin light polypeptide 6 | | 10 | -1.766841827 | | <0.001 | <0.001 |
| P49961 | ENTP1 – Ectonucleoside triphosphate diphosphohydrolase 1 | | 9 | -1.770423909 | | <0.001 | <0.001 |
| P14649 | MYL6B – Myosin light chain 6B | | 9 | -1.777243042 | | <0.001 | <0.001 |
| P05546 | HEP2 – Heparin cofactor 2 | | 16 | -1.778561033 | | <0.001 | <0.001 |
| P01591 | IGJ – Immunoglobulin J chain | | 8 | -1.784962119 | | <0.01 | <0.01 |
| Q93052 | LPP – Lipoma-preferred partner | | 29 | -1.792208928 | | <0.001 | <0.001 |
| O00159 | MYO1C – Unconventional myosin-Ic | | 49 | -1.79914756 | | <0.001 | <0.001 |
| P00739 | HPTR – Haptoglobin-related protein | | 7 | -1.807881632 | | <0.01 | <0.01 |
| Q6UWY5 | OLFL1 – Olfactomedin-like protein 1 | | 13 | -1.813201967 | | <0.001 | <0.001 |
| Q53TN4 | CYBR1 – Cytochrome b reductase 1 | | 3 | -1.82094853 | | <0.001 | <0.001 |
| P09455 | RET1 – Retinol-binding protein 1 | | 9 | -1.828235131 | | <0.001 | <0.001 |
| P02671 | FIBA – Fibrinogen alpha chain | | 60 | -1.879968132 | | <0.05 | <0.01 |
| P68133 | ACTS – Alpha-actin-1 | | 2 | -1.911461545 | | <0.001 | <0.001 |
| Q14315 | FLNC – Filamin-C | | 107 | -1.92873136 | | <0.001 | <0.001 |
| P02743 | SAMP – Serum amyloid P-component | | 11 | -1.943145428 | | <0.001 | <0.001 |
| P60981 | DEST – Destrin | | 12 | -1.967869924 | | <0.001 | <0.001 |
| P10301 | RRAS – Ras-related protein R-Ras | | 14 | -1.986053122 | | <0.001 | <0.001 |
| P01023 | A2MG – Alpha-2-macroglobulin | | 75 | -2.00585659 | | <0.001 | <0.001 |
| Q68CZ2 | TENS3 – Tensin-3 | | 20 | -2.008561926 | | <0.001 | <0.001 |
| P07738 | PMGE – Bisphosphoglycerate mutase | | 11 | -2.021951202 | | <0.001 | <0.001 |
| O75368 | SH3L1 – SH3 domain-binding glutamic acid-rich-like protein 1 | | 8 | -2.022416573 | | <0.001 | <0.001 |
| P02768 | ALBU – Albumin | | 100 | -2.027978309 | | <0.001 | <0.001 |
| P18206 | VINC – Vinculin | | 82 | -2.036145639 | | <0.001 | <0.001 |
| P06753 | TPM3 – Tropomyosin alpha-3 chain | | 36 | -2.048250457 | | <0.001 | <0.001 |
| P69891 | HBG1 – Haemoglobin subunit gamma-1 | | 7 | -2.075686751 | | <0.001 | <0.001 |
| Q8WU39 | MZB1 – Marginal zone B- and B1-cell-specific protein | | 8 | -2.080293581 | | <0.01 | <0.01 |
| Q05707 | COEA1 – Collagen alpha-1(XIV) chain | | 68 | -2.09873457 | | <0.001 | <0.001 |
| P21291 | CSRP1 – Cysteine and glycine-rich protein 1 | | 13 | -2.1514253 | | <0.001 | <0.001 |
| P13716 | HEM2 – Delta-aminolevulinic acid dehydratase | | 15 | -2.180358021 | | <0.01 | <0.01 |
| P21333 | FLNA – Filamin-A | | 169 | -2.187590377 | | <0.001 | <0.001 |
| P02647 | APOA1 – Apolipoprotein A-I | | 35 | -2.18857284 | | <0.001 | <0.001 |
| P07585 | PGS2 – Decorin | | 26 | -2.206845035 | | <0.001 | <0.001 |
| P08294 | SODE – Extracellular superoxide dismutase | | 12 | -2.218963629 | | <0.001 | <0.001 |
| Q86Y46 | K2C73 – Keratin, type II cytoskeletal 73 | | 3 | -2.230272282 | | <0.001 | <0.001 |
| P55268 | LAMB2 – Laminin subunit beta-2 | | 58 | -2.231997226 | | <0.001 | <0.001 |
| P39059 | COFA1 – Collagen alpha-1(XV) chain | | 15 | -2.26081612 | | <0.001 | <0.001 |
| O15230 | LAMA5 – Laminin subunit alpha-5 | | 84 | -2.279449202 | | <0.001 | <0.001 |
| Q9HBL0 | TENS1 – Tensin-1 | | 46 | -2.299559932 | | <0.001 | <0.001 |
| Q13425 | SNTB2 – Beta-2-syntrophin | | 13 | -2.41437426 | | <0.001 | <0.001 |
| Q15746 | MYLK – Myosin light chain kinase | | 48 | -2.437637008 | | <0.001 | <0.001 |
| P02654 | APOC1 – Apolipoprotein C-I | | 4 | -2.463745607 | | <0.05 | <0.05 |
| Q9NR12 | PDLI7 – PDZ and LIM domain protein 7 | | 21 | -2.514497229 | | <0.001 | <0.001 |
| P62736 | ACTA – Actin, aortic smooth muscle | | 21 | -2.608621477 | | <0.001 | <0.001 |
| P12277 | KCRB – Creatine kinase B-type | | 21 | -2.779624442 | | <0.001 | <0.001 |
| P02766 | TTR – Transthyretin | | 11 | -2.796960584 | | <0.001 | <0.001 |
| P09493 | TPM1 – Tropomyosin alpha-1 chain | | 25 | -2.832043183 | | <0.001 | <0.001 |
| P07951 | TPM2 – Tropomyosin beta chain | | 26 | -2.841326704 | | <0.001 | <0.001 |
| Q16853 | AOC3 – Membrane primary amine oxidase | | 21 | -2.882510929 | | <0.001 | <0.001 |
| P02652 | APOA2 – Apolipoprotein A-II | | 7 | -2.910543359 | | <0.001 | <0.001 |
| P34949 | MPI – Mannose-6-phosphate isomerase | | 7 | -3.016282551 | | <0.001 | <0.001 |
| Q9BX66 | SRBS1 – Sorbin and SH3 domain-containing protein 1 | | 35 | -3.046542825 | | <0.001 | <0.001 |
| Q01995 | TAGL – Transgelin | | 31 | -3.075365695 | | <0.001 | <0.001 |
| Q99459 | CDC5L – Cell division cycle 5-like protein | | 17 | -3.092589754 | | <0.01 | <0.01 |
| P35749 | MYH11 – Myosin-11 | | 231 | -3.238178757 | | <0.001 | <0.001 |
| P32119 | PRDX2 – Peroxiredoxin-2 | | 17 | -3.29406503 | | <0.01 | <0.01 |
| P20231 | TRYB2 – Tryptase beta-2 | | 16 | -3.371269387 | | <0.001 | <0.001 |
| Q15661 | TRYB1 – Tryptase alpha/beta-1 | | 16 | -3.371269387 | | <0.001 | <0.001 |
| Q03135 | CAV1 – Caveolin-1 | | 9 | -3.408389459 | | <0.001 | <0.001 |
| P68871 | HBB – Haemoglobin subunit beta | | 22 | -3.465642881 | | <0.01 | <0.01 |
| Q6NZI2 | CAVN1 – Caveolae-associated protein 1 | | 19 | -3.521064707 | | <0.001 | <0.001 |
| P11277 | SPTB1 – Spectrin beta chain | | 86 | -3.671171173 | | <0.01 | <0.01 |
| P20774 | MIME – Mimecan | | 23 | -3.70936941 | | <0.001 | <0.001 |
| Q9NZN4 | EHD2 – EH domain-containing protein 2 | | 36 | -3.733255327 | | <0.001 | <0.001 |
| P69905 | HBA – Haemoglobin subunit alpha | | 20 | -3.769500318 | | <0.01 | <0.01 |
| P51911 | CNN1 – Calponin-1 | | 27 | -3.789798779 | | <0.001 | <0.001 |
| P24844 | MYL9 – Myosin regulatory light polypeptide 9 | | 6 | -3.939013593 | | <0.001 | <0.001 |
| P02042 | HBD – Haemoglobin subunit delta | | 10 | -4.021552107 | | <0.01 | <0.01 |
| P00918 | CAH2 – Carbonic anhydrase 2 | | 18 | -4.035396298 | | <0.01 | <0.01 |
| O60271 | JIP4 – C-Jun-amino-terminal kinase-interacting protein 4 | | 24 | -4.346847515 | | <0.001 | <0.001 |
| P02730 | B3AT – Band 3 anion transport protein | | 23 | -4.674344703 | | <0.01 | <0.01 |
| P00915 | CAH1 – Carbonic anhydrase 1 | | 19 | -4.723264666 | | <0.01 | <0.01 |
| P17661 | DESM – Desmin | | 53 | -4.845020339 | | <0.001 | <0.001 |

Table S11: All Identified Dysregulated Proteins in GBC Compared to BBP Plasma

| Protein Group | Protein Name | No. of Unique Peptides | Average Log_2_ Ratio | p-value | q-value |
| --- | --- | --- | --- | --- | --- |
| P02741 | CRP – C-reactive protein | 6 | 2.650327304 | <0.001 | <0.001 |
| P02750 | A2GL – Leucine-rich alpha-2-glycoprotein | 12 | 1.239250891 | <0.001 | <0.001 |
| P18428 | LBP – Lipopolysaccharide-binding protein | 10 | 1.15874307 | <0.001 | <0.001 |
| P01011 | AACT – Alpha-1-antichymotrypsin | 24 | 1.07655644 | <0.001 | <0.001 |
| Q06033 | ITIH3 – Inter-alpha-trypsin inhibitor heavy chain H3 | 18 | 1.05262098 | <0.001 | <0.001 |
| P02763 | A1AG1 – Alpha-1-acid glycoprotein 1 | 13 | 1.029932151 | <0.001 | <0.001 |
| P0DJI8 | SAA1 – Serum amyloid A-1 protein | 5 | 1.010432073 | <0.001 | <0.001 |
| P01009 | A1AT – Alpha-1-antitrypsin | 32 | 0.933125857 | <0.001 | <0.001 |
| Q9Y6R7 | FCGBP – IgGFc-binding protein | 12 | 0.843403272 | <0.001 | <0.001 |
| P01833 | PIGR – Polymeric immunoglobulin receptor | 16 | 0.727327957 | <0.001 | <0.001 |
| P02776 | PLF4 – Platelet factor 4 | 4 | 0.704884661 | <0.05 | <0.01 |
| P02649 | APOE – Apolipoprotein E | 18 | 0.691933152 | <0.001 | <0.001 |
| P03952 | KLKB1 – Plasma kallikrein | 23 | -0.681121022 | <0.001 | <0.001 |
| P01700 | LV147 – Immunoglobulin lambda variable 1-47 | 2 | -0.70219583 | <0.001 | <0.001 |
| P02765 | FETUA – Fetuin-A | 10 | -0.711729896 | <0.001 | <0.001 |
| P02790 | HEMO – Hemopexin | 26 | -0.739818383 | <0.001 | <0.001 |
| P02753 | RET4 – Retinol-binding protein 4 | 9 | -0.748467682 | <0.001 | <0.001 |
| P06396 | GELS – Gelsolin | 26 | -0.773859647 | <0.001 | <0.001 |
| P29622 | KAIN – Kallistatin | 12 | -0.815267411 | <0.001 | <0.001 |
| P17936 | IBP3 – Insulin-like growth factor-binding protein 3 | 2 | -0.889184722 | <0.001 | <0.001 |
| Q9UGM5 | FETUB – Fetuin-B | 6 | -0.919334394 | <0.001 | <0.001 |
| P60709 | ACTB – Actin, cytoplasmic 1 | 16 | -0.94638678 | <0.001 | <0.001 |
| P05452 | TETN – Tetranectin | 7 | -0.951549061 | <0.001 | <0.001 |
| P02647 | APOA1 – Apolipoprotein A-I | 30 | -0.957554639 | <0.001 | <0.001 |
| P68871 | HBB – Haemoglobin subunit beta | 15 | -0.977033982 | <0.001 | <0.001 |
| P49908 | SEPP1 – Selenoprotein P | 3 | -0.990550309 | <0.001 | <0.001 |
| P69905 | HBA – Haemoglobin subunit alpha | 8 | -1.088464667 | <0.001 | <0.001 |
| O14791 | APOL1 – Apolipoprotein L1 | 10 | -1.094391403 | <0.001 | <0.001 |
| Q96PD5 | PGRP2 – Peptidoglycan recognition protein 2 | 13 | -1.125975222 | <0.001 | <0.001 |
| P02766 | TTR – Transthyretin | 10 | -1.147028578 | <0.001 | <0.001 |
| P20742 | PZP – Pregnancy zone protein | 35 | -1.170088816 | <0.001 | <0.001 |
| P27169 | PON1 – Serum paraoxonase/arylesterase 1 | 9 | -1.263906253 | <0.001 | <0.001 |
| P02652 | APOA2 – Apolipoprotein A-II | 7 | -1.474317396 | <0.001 | <0.001 |

Table S12: Common Dysregulated Proteins Across Group Comparisons with Relative Average Log_2_ Fold Change

| Common Proteins  in GBC/Normal & GBC/GD | Average Log_2_ Fold Change | | Common Proteins in  GBC/GD & GBC/BBP | Average Log_2_ Fold Change | |  |
| --- | --- | --- | --- | --- | --- | --- |
|  | **GBC/Norm** | **GBC/GD** |  | **GBC/GD** | **GBC/BBP** |  |
| CAH2 – Carbonic anhydrase 2 | -3.359399413 | -4.035396298 | **APOA1 – Apolipoprotein A-I** | -2.18857284 | -0.957554639 |  |
| JIP4 – ****C-Jun-amino-terminal kinase-interacting protein 4**** | -4.856406351 | -4.346847515 | **APOA2 – Apolipoprotein A-II** | -2.910543359 | -1.474317396 |  |
| VTNC – Vitronectin | 3.915829431 | 1.508175969 | **RET4 – Retinol-binding protein 4** | -1.68830487 | -0.748467682 |  |
| H2A1B; H2A3; H2A1C – Histone H2B type 1-A; Histone H2A type 3; Histone H2A type 1-C | 3.601816369 | -1.597290839 | **TTR – Transthyretin** | -2.796960584 | -1.147028578 |  |
| TPM2 – Tropomyosin beta chain | -2.980807556 | -2.841326704 | **HEMO – Hemopexin** | -1.618488319 | -0.739818383 |  |
| TSP1 – Thrombospondin-1 | 3.251710872 | 3.003882122 | **HBB – Haemoglobin subunit beta** | -3.465642881 | -0.977033982 |  |
| TPM1 – Tropomyosin alpha-1 chain | -2.60007637 | -2.832043183 | **HBA – Haemoglobin subunit alpha** | -3.769500318 | -1.088464667 |  |
| RRAS – Ras-related protein R-Ras | -2.788038724 | -1.986053122 |  |  |  |  |
| PCNA – Proliferating cell nuclear antigen | 3.172973883 | 2.479580402 | **Common Proteins in GBC/Normal,**  **GBC/GD, & GBC/BBP** | **Average Log_2_ Fold Change** | | |
| RAC2 – Ras-related C3 botulinum toxin substrate 2 | 2.737986073 | 1.675119445 |  | **GBC/Normal** | **GBC/GD** | **GBC/BBP** |
| DESM – Desmin | -3.441316866 | -4.845020339 | **PIGR – Polymeric immunoglobulin receptor** | 2.898754081 | 2.196173212 | 0.727327957 |
| TYPH – Thymidine phosphorylase | 2.561671217 | 1.795493292 | **APOE – Apolipoprotein E** | 2.968525542 | 2.088429222 | 0.691933152 |
| TRYB2; TRYB1 – Tryptase beta-2; Tryptase alpha/beta-1 | -2.800721074 | -3.371269387 |  |  |  |  |
| MIME – Mimecan | -2.895872701 | -3.70936941 |  |  |  |  |
| FLNA – Filamin-A | -2.489748582 | -2.187590377 |  |  |  |  |
| TENA – Tenascin | 2.712643782 | 1.763552536 |  |  |  |  |
| MYL9 – Myosin regulatory light polypeptide 9 | -3.628772097 | -3.939013593 |  |  |  |  |
| GLYM – ****Serine hydroxymethyltransferase**** | 2.51078038 | 1.862362934 |  |  |  |  |
| MYH11 – Myosin-11 | -3.652336209 | -3.238178757 |  |  |  |  |
| CNN1 – Calponin-1 | -2.741320166 | -3.789798779 |  |  |  |  |
| RIDA – 2-iminobutanoate/2-iminopropanoate deaminase | 3.70928337 | 3.587142384 |  |  |  |  |
| AK1C2 – ****Aldo-keto reductase family 1 member C2**** | 3.848081936 | 4.953323214 |  |  |  |  |
| ACTA – Actin, aortic smooth muscle | -2.492619937 | -2.608621477 |  |  |  |  |
| TAGL – Transgelin | -3.372011937 | -3.075365695 |  |  |  |  |
| BDH – D-beta-hydroxybutyrate dehydrogenase | 2.900769273 | 3.638123672 |  |  |  |  |
| CAV1 – Caveolin-1 | -2.651299406 | -3.408389459 |  |  |  |  |
| POSTN – Periostin | 5.02066359 | 2.216168479 |  |  |  |  |
| CAVN1 – ****Caveolae-associated protein 1**** | -3.822693564 | -3.521064707 |  |  |  |  |
| DCXR – L-xylulose reductase | 2.983416149 | 2.844273258 |  |  |  |  |
| COCA1 – ****Collagen alpha-1(XII) chain**** | 4.004558467 | 3.374782172 |  |  |  |  |
| PARVB – Beta-parvin | -3.106309788 | -1.560212729 |  |  |  |  |
| EHD2 – EH domain-containing protein 2 | -3.475631992 | -3.733255327 |  |  |  |  |
| CPSM – Carbamoyl-phosphate synthase | 4.483369762 | 4.323381454 |  |  |  |  |
| Norm = Normal  GD = Gallstone Disease  GBC = Gallbladder Cancer  BBP = Benign Biliary Pathology | | | | | | |

Table S13: Non-Metastatic vs Metastatic Patients for GBC Plasma Dysregulated Proteins

| Protein Name | Non-metastatic Mean | Metastatic Mean | p-value |
| --- | --- | --- | --- |
| APOA1 – Apolipoprotein A-I | 1180.14 | 1497.55 | 0.1275 |
| APOA2 – Apolipoprotein A-II | 80.07 | 146.11 | 0.2348 |
| RET4 – Retinol-binding protein 4 | 26.12 | 20.39 | 0.5730 |
| TTR - Transthyretin | 205.75 | 197.60 | 0.7853 |
| HEMO - Hemopexin | 1033.80 | 1457.41 | 0.1474 |
| HBB – Haemoglobin subunit beta | 2196.72 | 1183.42 | 0.6761 |
| HBA – Haemoglobin subunit alpha | 697.44 | 379.40 | 0.7029 |
| PIGR – Polymeric immunoglobulin receptor | 39.21 | 35.96 | 0.7029 |
| APOE* – Apolipoprotein E | 926.01 | 552.41 | 0.0143* |
| CRP – C-reactive protein | 461.85 | 274.26 | 0.8990 |
| GELS – Gelsolin | 31.96 | 34.29 | 0.9855 |
| APOL1 – Apolipoprotein L1 | 11.40 | 11.52 | 0.6497 |
| A1AT – Alpha-1-antitrypsin | 40335.51 | 30706.32 | 0.1098 |
| AACT – Alpha-1-antichymotrypsin | 3379.44 | 2353.12 | 0.3526 |
| LV147 – Immunoglobulin lambda variable 1-47 | 84.55 | 53.27 | 0.5009 |
| A2GL – Leucine-rich alpha-2-glycoprotein | 705.06 | 498.34 | 0.2348 |
| A1AG1 – Alpha-1-acid glycoprotein 1 | 1982.38 | 1477.00 | 0.5244 |
| FETUA – Fetuin-A | 430.90 | 476.94 | 0.2348 |
| PLF4 – Platelet factor 4 | 11.28 | 3.52 | 0.1653 |
| KLKB1 – Plasma kallikrein | 27.03 | 26.95 | 0.7029 |
| TETN – Tetranectin | 4.28 | 5.17 | 0.4779 |
| SAA1 – Serum amyloid A-1 protein | 68.78 | 62.65 | 0.6995 |
| IBP3 – Insulin-like growth factor-binding protein 3 | 7.82 | 9.94 | 0.5358 |
| LBP – Lipopolysaccharide-binding protein | 49.98 | 24.60 | 0.3919 |
| PZP – Pregnancy zone protein | 17.63 | 23.37 | 0.3426 |
| PON1 – Serum paraoxonase/arylesterase 1 | 38.29 | 52.04 | 0.1184 |
| KAIN – Kallistatin | 6.14 | 5.91 | 0.8079 |
| SEPP1 – Selenoprotein P | 2.83 | 3.36 | 0.5855 |
| ACTB – Actin, cytoplasmic 1 | 10.81 | 22.42 | 0.0940 |
| ITIH3* – Inter-alpha-trypsin inhibitor heavy chain H3 | 94.64 | 60.16 | 0.0173* |
| PGRP2 – Peptidoglycan recognition protein 2 | 7.39 | 7.16 | 0.4337 |
| FETUB – Fetuin-B | 24.49 | 27.70 | 0.3460 |
| FCGBP – IgGFc-binding protein | 3.68 | 3.31 | 0.7606 |

*This indicates the only two proteins which were significantly dysregulated in non-metastatic vs metastatic patients
